# LRRC8A Inhibition Overcomes Chemoresistance by Downregulating MRP3 and CYP3A4 in the 3D Spheroid Model of Human Breast Cancer Cells

**DOI:** 10.3390/ijms27062646

**Published:** 2026-03-13

**Authors:** Ryo Otsuka, Junko Kajikuri, Miki Matsui, Hiroaki Kito, Ayano Kitahara, Hinako Mitsui, Yohei Yamaguchi, Tomoka Hisada, Tatsuya Toyama, Susumu Ohya

**Affiliations:** 1 Department of Pharmacology, Graduate School of Medical Sciences, Nagoya City University, Nagoya 467-8601, Japan; c241905@ed.nagoya-cu.ac.jp (R.O.); kajikuri@med.nagoya-cu.ac.jp (J.K.); c241739@ed.nagoya-cu.ac.jp (M.M.); kito@med.nagoya-cu.ac.jp (H.K.); kita.aya@med.nagoya-cu.ac.jp (A.K.); c212057@ed.nagoya-cu.ac.jp (H.M.); y_yamagu@med.nagoya-cu.ac.jp (Y.Y.); 2 Department of Breast Surgery, Graduate School of Medical Sciences, Nagoya City University, Nagoya 467-8601, Japant.toyama@med.nagoya-cu.ac.jp (T.T.)

**Keywords:** breast cancer, cancer stemness, LRRC8A, chemoresistance, MRP3, CYP3A4, NRF2

## Abstract

Leucine-rich repeat-containing 8A (LRRC8A; also known as SWELL1), the essential subunit of volume-regulated anion channels (VRACs), is amplified in multiple malignancies and has been implicated in tumor progression and therapeutic resistance. Three-dimensional (3D) cancer spheroids have been well-established as in vitro models that recapitulate characteristics of tumor stemness and intrinsic drug resistance. In the present study, spheroid formation in human breast cancer cell lines, YMB-1 and MDA-MB-468, conferred resistance to multiple anticancer drugs, including doxorubicin (DOX), gemcitabine (GEM), and 5-fluorouracil (5-FU), thereby mimicking the characteristic properties of breast cancer stem-like cells. LRRC8A expression was upregulated in 3D spheroids compared with adherent 2D monolayers, and its pharmacological inhibition induced membrane hyperpolarization accompanied by intracellular Cl^−^ accumulation. Inhibition of LRRC8A significantly sensitized spheroids to DOX, GEM, and 5-FU. Spheroid formation increased the expression of multidrug resistance-related protein 3 (MRP3) and the drug-metabolizing enzyme cytochrome P450 3A4 (CYP3A4), whereas LRRC8A inhibition suppressed their expression. The transcriptional upregulation of MRP3 and CYP3A4 was mediated through the NRF2–CEBPB/D transcriptional axis. Collectively, these findings suggest that LRRC8A inhibition may represent a therapeutic strategy to overcome chemoresistance by repressing MRP3 and/or CYP3A4 expression in breast cancer stem cells.

## 1. Introduction

3D cancer spheroid cultures have emerged as physiologically relevant in vitro models that closely mimic the microenvironmental characteristics of solid tumors, including cancer stemness and drug resistance [1]. In breast cancer, 3D spheroids recapitulate key characteristics of cancer stem-like cells, such as reduced proliferative activity and increased expression of stemness-associated transcription factors [2]. These spheroids exhibit intrinsic resistance to multiple chemotherapeutic agents, providing a valuable platform for elucidating the molecular mechanisms underlying chemoresistance. Accumulating evidence further indicates that ion channels play critical roles in the development and maintenance of chemoresistance across diverse cancer types [3].

The volume-regulated anion channel (VRAC) plays a pivotal role in maintaining cellular homeostasis by controlling Cl^−^ and osmolyte fluxes in response to changes in cell volume [4]. Recent studies have identified leucine-rich repeat-containing 8 (LRRC8) as an essential component of the VRAC. Functional VRACs are formed by the obligatory LRRC8A subunit in combination with at least one of its paralogs (LRRC8B-E), generating heteromeric assemblies such as 8A/C, 8A/D, and 8A/E [5]. Beyond its canonical role in cell volume regulation, accumulating evidence suggests that LRRC8A contributes to cancer pathophysiology [6]. Amplification and overexpression of LRRC8A have been reported in multiple tumor types and correlate with increased proliferation, metastatic potential, and poor prognosis [7]. Moreover, LRRC8A has been implicated in modulating cellular sensitivity to a wide range of anticancer agents [8]. Thus, LRRC8A represents a potential oncogenic driver and promising therapeutic target in cancer biology. However, its functional roles in 3D cancer spheroid systems remain largely unexplored.

Breast cancer is a highly heterogeneous disease, and subpopulations with cancer stem cell-like properties exhibit intrinsic resistance to anticancer drugs [9,10]. Despite significant therapeutic advances, chemoresistance remains a major clinical challenge that limits treatment efficacy and promotes disease recurrence and progression. The acquisition of chemoresistance in breast cancer involves multiple mechanisms, including the overexpression of ATP-binding cassette (ABC) multidrug efflux transporters, such as multidrug resistance proteins (MDRs) and MDR-related proteins (MRPs), as well as cytochrome P450 drug-metabolizing enzymes (CYPs), which collectively reduce intracellular drug concentrations and diminish the effectiveness of chemotherapeutic agents [11,12]. In cancer stem cells, constitutive activation of the oncogenic PI3K/AKT and MEK/ERK signaling pathways plays a critical role in promoting chemoresistance [13,14]. Notably, LRRC8A has been shown to participate in the activation of these pathways in cancer cells [6,15,16].

Doxorubicin (DOX), an anthracycline antibiotic, is widely used in breast cancer chemotherapy [17]. Among CYPs, CYP3A4, along with CYP2D6, CYP2B6, and CYP1B1 to a lesser extent, plays a principal role in DOX metabolism [18]. Likewise, 5-fluorouracil (5-FU) and gemcitabine (GEM) are commonly used chemotherapeutic agents in breast cancer treatment [17], and resistance to these drugs has also been associated with CYP3A4 activity [19]. Among the MRPs, MRP3 and MRP5 contribute to resistance to DOX, GEM, and/or 5-FU in cancer cells [20,21,22].

Nuclear factor-erythroid 2-related factor 2 (NRF2) is known as a crucial regulator of oxidative stress, and NRF2 dissociates from Kelch-like ECH-associated protein 1, KEAP1, and subsequently translocates into the nucleus [23,24]. CCAAT/enhancer-binding protein (C/EBP and CEBP) isoforms are a family of multifunctional basic leucine zipper transcription factors [25]. We previously reported the transcriptional expression of interleukin-8 (IL-8), IL-10, and C-C motif chemokine ligand 2 (CCL2) were suppressed in M_2_-polarized macrophages following treatment with LRRC8A inhibition via the NRF2-CEBPB signaling axis [26,27].

The present study was designed to elucidate the functional role of LRRC8A in chemoresistance using a physiologically relevant 3D spheroid model of human breast cancer cells and to define the molecular mechanisms underlying drug resistance and established LRRC8A inhibition as a rational therapeutic strategy for overcoming chemoresistance in breast cancer. To achieve this objective, we adopted a stepwise experimental strategy consisting of: (1) functional evaluation of LRRC8A expression and channel activity in 3D spheroids; (2) assessment of chemoresistance profiles and their reversal following pharmacological and genetic inhibition of LRRC8A; (3) identification of downstream mediators of drug resistance; and (4) mechanistic dissection of the transcriptional regulatory axis linking LRRC8A to effector gene expression.

## 2. Results

### 2.1. Functional Expression of LRRC8A in a Three-Dimensional (3D) Spheroid Model of Human Breast Cancer YMB-1 Cells

**Figure 1:**
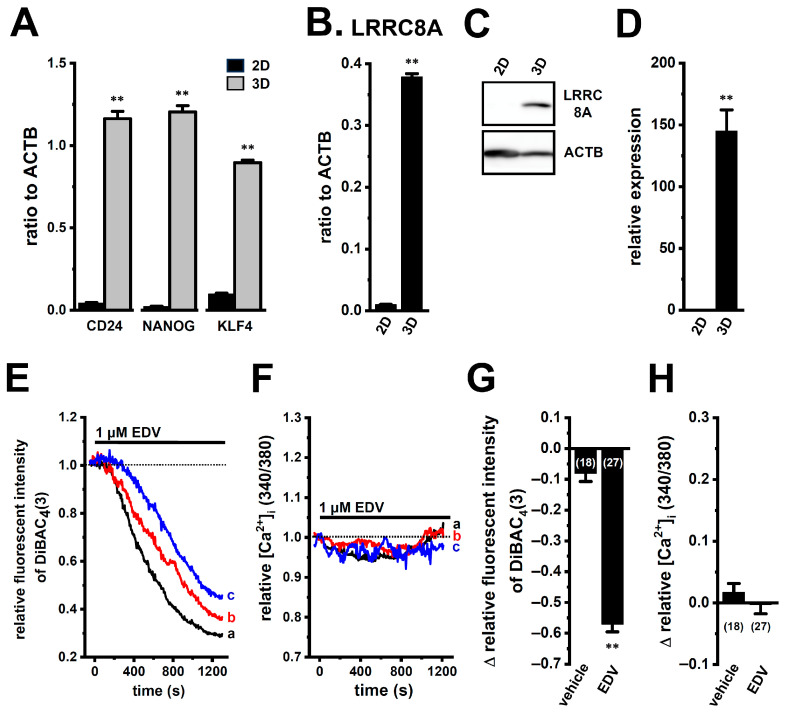
Comparison of LRRC8A channel expression between 2D monolayers and 3D spheroids of human breast cancer YMB-1 cells and functional characteristics of LRRC8A in cells isolated from 3D spheroids. (**A**,**B**): Real-time PCR examination of the CD24, NANOG, KLF4 (**A**), and LRRC8A (**B**) transcripts in 2D monolayers (‘2D’) and 3D spheroids (‘3D’) of YMB-1 cells. Expression levels are shown as a ratio to ACTB (*n* = 4). (**C**,**D**): LRRC8A protein expression in the lipid raft-enriched protein lysates of ‘2D’ and ‘3D’ groups. Blots were probed with anti-LRRC8A (upper panel), and anti-ACTB (lower panel) antibodies (**C**). Summarized results were obtained as the optical density of LRRC8A and ACTB band signals (**D**). After compensation for the optical density of the LRRC8A protein band signal with that of the ACTB signal, the optical density in ‘2D’ was expressed as 1.0 (*n* = 4). (**E**–**H**): Simultaneous measurement of changes in membrane potential (**E**) and intracellular Ca^2+^ concentration ([Ca^2+^]_i_) (**F**) in three different cells [black (a), red (b), and blue (c)], following the application of the VRAC inhibitor, endovion (EDV, 1 μM), using DiBAC_4_(3) and Fura 2, respectively. The relative time courses of changes in fluorescence intensities (1.0 at time 0 s) from isolated cells are shown. Summarized results of EDV (1 μM)-induced hyperpolarizing responses (**G**) and changes in [Ca^2+^]_i_ (**H**) at 20 min. Numbers used for experiments are shown in parentheses. **: *p* < 0.01 vs. ‘2D’ and vehicle control.

3D spheroids of YMB-1 cells were generated using the ultra-low attachment culture plates (see ‘Section 4.2’). Seven days after seeding, compact spheroidal aggregates were formed (Supplementary Figure S1A). The cancer stemness marker CD24 and the stemness-associated transcriptional factors NANOG and KLF4 were markedly overexpressed in 3D spheroids compared with the 2D monolayers (*n* = 4 for each, *p* < 0.01) (Figure 1A).

We next compared LRRC8A gene and protein expression between the 2D monolayers and 3D spheroids by quantitative, real-time PCR and Western blotting. Both LRRC8A transcripts and proteins were markedly upregulated in 3D spheroids (*n* = 4, *p* < 0.01 vs. 2D) (Figure 1B–D). Functional VRACs require heteromeric assembly of LRRC8A with LRRC8B-E subunits at the plasma membrane [28]. We found that LRRC8B-E transcripts were concomitantly upregulated during spheroid formation (Supplementary Figure S1B–E).

Endovion (EDV) is a potent anion channel inhibitor that blocks the VRAC activity (half-maximal inhibitory concentration, IC_50_ = approx. 0.5 μM) and inhibits both LRRC8 and Ca^2+^-activated Cl^−^ channels (anoctamin 1/2, ANO1/2). The application of 1 μM EDV to cells acutely isolated from 3D spheroids induced large hyperpolarizing responses (Figure 1E,G) accompanied by intracellular Cl^−^ accumulation, whereas no significant changes in intracellular Ca^2+^ concentration ([Ca^2+^]_i_) were observed (Figure 1F,H). Conversely, ANO1, but not ANO2, was predominantly expressed in 3D spheroids (Supplementary Figure S2A), and the application of 10 μM ANO1-IN-1, a selective ANO1 inhibitor (IC_50_ = approx. 3 μM), evoked hyperpolarization accompanied by a significant rise in [Ca^2+^]_i_ (Supplementary Figure S2B–E). The application of ionomycin (1 μM), a Ca^2+^ ionophore, evoked an instantaneous increase in [Ca^2+^]_i_ (1.69 ± 0.07-fold, *n* = 11).

### 2.2. Overcoming Chemoresistance by LRRC8A Inhibition in YMB-1 3D Spheroids

**Figure 2:**
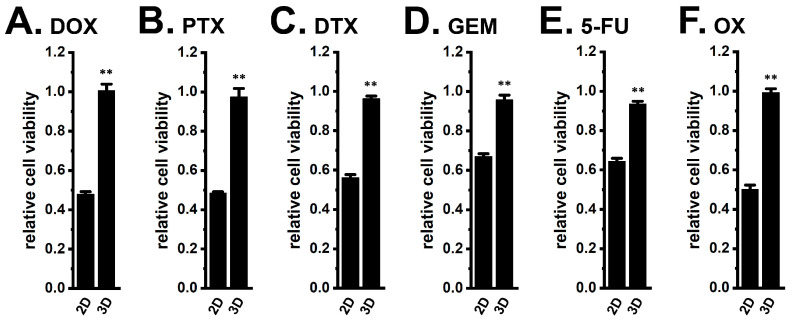
Effects of chemotherapeutic agents on the viability of 2D monolayers and 3D spheroids of YMB-1 cells. (**A**–**F**): Effects of the treatment with 1 μM doxorubicin (DOX) (**A**), 0.1 μM paclitaxel (PTX) (**B**), 0.1 μM docetaxel (DTX) (**C**), 10 μM gemcitabine (GEM) (**D**), 10 μM 5-fluorouracil (5-FU) (**E**), and 1 μM oxaliplatin (OX) (**F**) for 48 h in 2D monolayers (‘2D’) and 3D spheroids (‘3D’) using the WST-1 assay (*n* = 5). Cell viability in the untreated group was expressed as 1.0. **: *p* < 0.01 vs. ‘2D’.

The optimal seeding density for cell viability assay was determined as previously reported in several cancer cell lines [29]. As shown in Figure 2A,B, 3D spheroids exhibited resistance to DOX (1 μM) and paclitaxel (PTX, 100 nM), the most widely used chemotherapy drugs, following exposure for 48 h (*n* = 5, *p* < 0.01 vs. 2D). Similar resistance was observed for docetaxel (DTX, 100 nM), GEM (10 μM), 5-FU (10 μM), and oxaliplatin (OX, 1 μM), all clinically used in breast cancer therapy [17] (Figure 2C–F). Concentration–response profiles for all agents in 2D and 3D cultures are summarized in Supplementary Figure S3.

**Figure 3:**
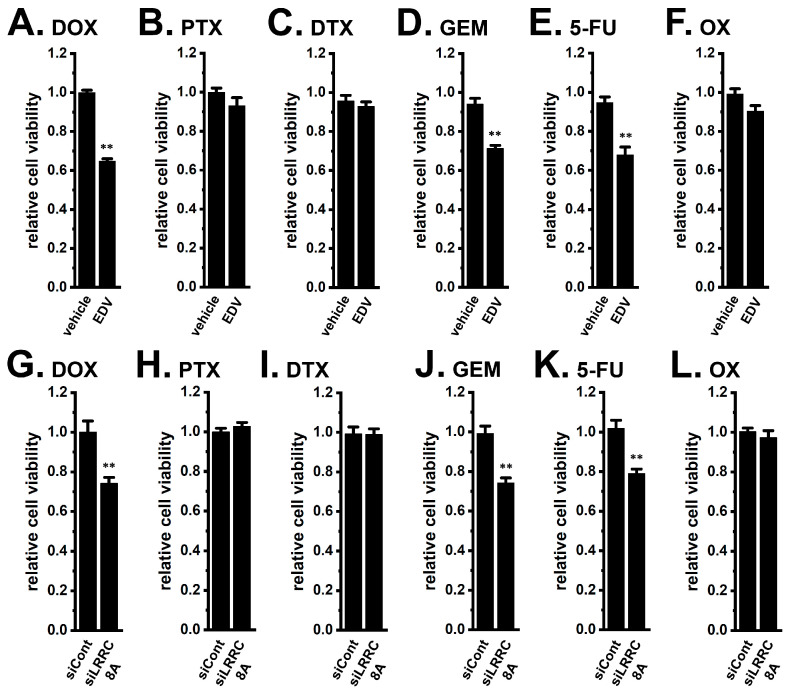
Effects of the pharmacological inhibition of VRAC and siRNA-mediated knockdown of LRRC8A on resistance to chemotherapeutic agents in YMB-1 spheroids. (**A**–**F**): Effects of the treatment with 1 μM DOX (**A**), 0.1 μM PTX (**B**), 0.1 μM DTX (**C**), 10 μM GEM (**D**), 10 μM 5-FU (**E**), and 1 μM OX (**F**) for 48 h on the cell viability of YMB-1 spheroids pre-treated with vehicle (0.1% DMSO) and 10 μM EDV for 24 h (*n* = 5). (**G**–**L**): Effects of the treatment with DOX (**G**), PTX (**H**), DTX (**I**), GEM (**J**), 5-FU (**K**), and OX (**L**) for 48 h on the cell viability of YMB-1 spheroids transfected with negative control siRNA (siCont) and human LRRC8A siRNA (siLRRC8A) (*n* = 5). Cell viability in the untreated group was expressed as 1.0. **: *p* < 0.01 vs. vehicle-treated and siCont-transfected groups.

Pre-treatment of the YMB-1 3D spheroids with 10 μM EDV for 24 h significantly reversed resistance to DOX, GEM, and 5-FU (*n* = 5, *p* < 0.01) (Figure 3A,D,E), whereas resistance to PTX, DTX, and OX remained unchanged (*n* = 5, *p* > 0.05) (Figure 3B,C,F). Consistently, siRNA-mediated inhibition of LRRC8A (Supplementary Figures S4A and S5A) similarly restored DOX, GEM, and 5-FU sensitivity (*n* = 5, *p* < 0.01) (Figure 3G,J,K) without affecting responses to PTX, DTX, or OX (*n* = 5, *p* > 0.05) (Figure 3H,I,L). Neither EDV treatment nor siLRRC8A transfection affected basal viability [0.970 ± 0.019 and 0.977 ± 0.022 in arbitrary units (a.u.), respectively, *n* = 5, *p* > 0.05]. On the other hand, neither pharmacological (with 10 μM ANO1-IN-1) nor siRNA-mediated inhibition of ANO1 (Supplementary Figures S4B and S5B) altered resistance to DOX or PTX (*n* = 5, *p* > 0.05) (Supplementary Figure S6A–D).

### 2.3. Upregulation of Drug Efflux Transporter MRP3 and Drug Metabolizing Enzyme CYP3A4 During Spheroid Formation and Their Downregulation by LRRC8A Inhibition in YMB-1 3D Spheroids

**Figure 4:**
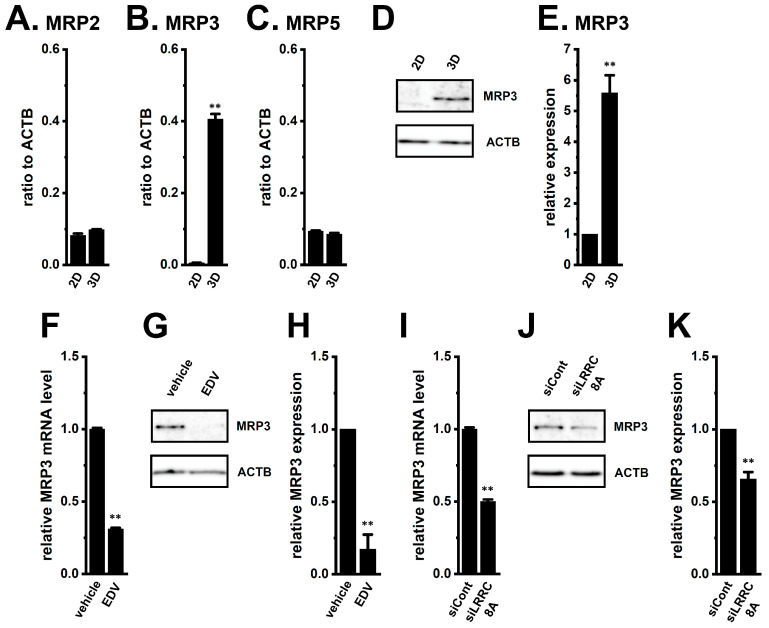
Comparison of gene and/or protein expression levels of MRP isoforms between 2D monolayers and 3D spheroids of YMB-1 cells and effects of the LRRC8A inhibition on MRP3 expression levels in 3D spheroids. (**A**–**C**): Real-time PCR examination of MRP2 (**A**), MRP3 (**B**), and MRP5 (**C**) in ‘2D’ and ‘3D’groups. Expression levels are shown as a ratio to ACTB (*n* = 4). (**D**,**E**): Protein expression of MRP3 in the lipid raft-enriched protein lysates of ‘2D’ and ‘3D’ groups. Blots were probed with anti-MRP3 (**D**) (upper panel) and anti-ACTB (lower panel) antibodies. Summarized results were obtained as the optical density of MRP3 and ACTB band signals. After compensating for the optical density of the MRP3 protein band signal, the optical density in ‘2D’ was expressed as 1.0 (*n* = 4) (**E**). (**F**,**I**): Real-time PCR examination of MRP3 in 3D spheroids treated with vehicle and 10 μM EDV for 12 h (**F**) and transfected with siCont and siLRRC8A (**I**). After normalization to ACTB levels, MRP3 mRNA expression levels in the vehicle-treated and siCont-transfected groups were expressed as 1.0 (*n* = 4). (**G**,**H**,**J**,**K**): Protein expression of MRP3 in 3D spheroids treated with vehicle and 10 μM EDV for 24 h (**G**,**H**) and transfected with siCont and siLRRC8A (**J**,**K**). Blots were probed with anti-MRP3 (upper panel) and anti-ACTB (lower panel) antibodies. Summarized results were obtained as the optical density of MRP3 and ACTB band signals. After compensating for the optical densities of the MRP3 protein band signal, the optical density in the vehicle-treated and siCont-transfected groups was expressed as 1.0 (*n* = 4) (**H**,**K**). **: *p* < 0.01 vs. ‘2D’, vehicle control, and siCont.

Among the 13 ABC transporter genes examined (MDR1, MDR3, ABCB5, MRP1-7, ABCB10, and ABCG1-2), MRP2, MRP3, and MRP5 were highly expressed in YMB-1 3D spheroids (Figure 4A–C). The MRP3 gene and protein levels markedly increased during spheroid formation (*n* = 4, *p* < 0.01) (Figure 4B,D,E), whereas other isoforms were minimally expressed (< 0.02 a.u. to ACTB, *n* = 4). EDV (10 μM) significantly reduced MRP3 expression levels at both mRNA (12 h) and protein (24 h) levels (*n* = 4, *p* < 0.01) (Figure 4F–H). Similar results were obtained by siLRRC8A transfection (*n* = 4, *p* < 0.01) (Figure 4I–K). Anoctamin 1 (ANO1) inhibition had no significant effects (*n* = 4, *p* > 0.05) (Supplementary Figures S4B and S7A,B). Given prior evidence implicating MRP3 in the efflux of DOX, GEM, and 5-FU [20,21,30], its suppression by LRRC8A inhibition likely contributes to restored sensitivity to DOX, GEM, and 5-FU in YMB-1 3D spheroids.

**Figure 5:**
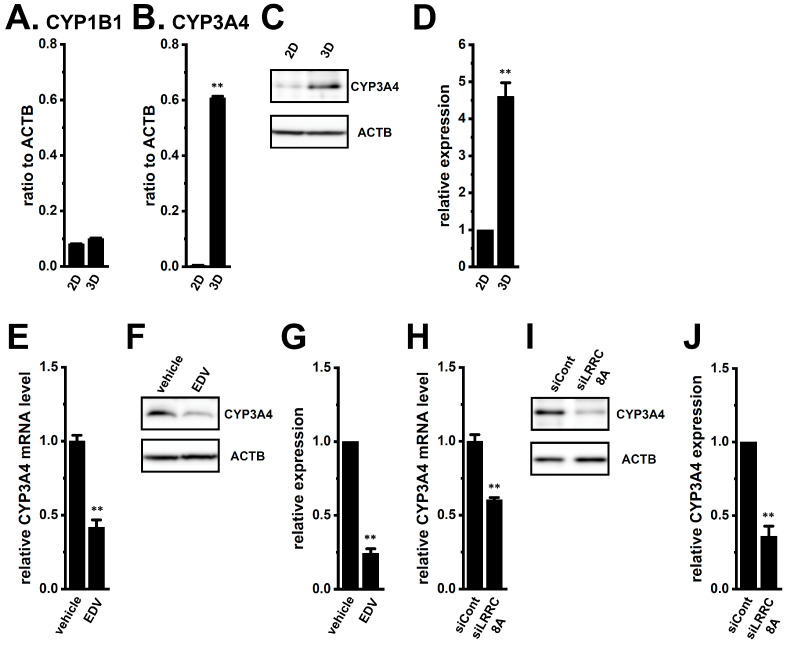
Comparison of gene and/or protein expression levels of CYP isoforms between 2D monolayers and 3D spheroids of YMB-1 cells and effects of the LRRC8A inhibition on CYP3A4 expression levels in 3D spheroids. (**A**,**B**): Real-time PCR examination of CYP1B1 (**A**) and CYP3A4 (**B**) in ‘2D’ and ‘3D’groups. Expression levels are shown as a ratio to ACTB (*n* = 4). (**C**,**D**): Protein expression of CYP3A4 in ‘2D’ and ‘3D’ groups. Blots were probed with anti-CYP3A4 (**C**) (upper panel) and anti-ACTB (lower panel) antibodies. Summarized results were obtained as the optical density of CYP3A4 and ACTB band signals. After compensating for the optical density of the CYP3A4 protein band signal, the optical density in ‘2D’ was expressed as 1.0 (*n* = 4) (**D**). (**E**,**H**): Real-time PCR examination of CYP3A4 in 3D spheroids treated with vehicle and 10 μM EDV for 12 h (**E**) and transfected with siCont and siLRRC8A (**H**). After normalization to ACTB levels, CYP3A4 mRNA expression levels in the vehicle-treated and siCont-transfected groups were expressed as 1.0 (*n* = 4). (**F**,**G**,**I**,**J**): Protein expression of CYP3A4 in 3D spheroids treated with vehicle and 10 μM EDV for 24 h (**F**,**G**) and transfected with siCont and siLRRC8A (**I**,**J**). Blots were probed with anti-CYP3A4 (upper panel) and anti-ACTB (lower panel) antibodies. Summarized results were obtained as the optical density of CYP3A4 and ACTB band signals. After compensating for the optical densities of the CYP3A4 protein band signal, the optical density in the vehicle-treated and siCont-transfected groups was expressed as 1.0 (*n* = 4) (**G**,**J**). **: *p* < 0.01 vs. ‘2D’, vehicle control, and siCont.

CYP3A4, together with CYP2D6, CYP2B6, and CYP1B1 to a minor extent, plays a central role in DOX metabolism [18]. Among the several CYP isoforms examined (CYP2A6, 2A7, 2B6, 2C8, 2D6, 3A5, and 4Z1), CYP1B1 and CYP3A4 were dominantly expressed in 3D spheroids (Figure 5A,B), with CYP3A4 gene and protein levels markedly upregulated (*n* = 4, *p* < 0.01) (Figure 5B–D), whereas other isoforms were minimally expressed (<0.01 a.u., *n* = 4). LRRC8A inhibition with EDV (10 μM) significantly reduced CYP3A4 expression levels at both mRNA (12 h) and protein (24 h) levels (*n* = 4, *p* < 0.01) (Figure 5E–G). Similar results were obtained by siLRRC8A transfection (*n* = 4, *p* < 0.01) (Figure 5H–J). ANO1 inhibition had no significant effects (*n* = 4, *p* > 0.05) (Supplementary Figure S7C,D). As CYP3A4 contributes to the metabolism of DOX, GEM, and 5-FU [18,19], these findings suggest that LRRC8A inhibition attenuates resistance to them through CYP3A4 in YMB-1 3D spheroids.

### 2.4. Overcoming Chemoresistance by siRNA-Mediated Inhibition of MRP3 and CYP3A4 inYMB-1 3D Sphroids

**Figure 6:**
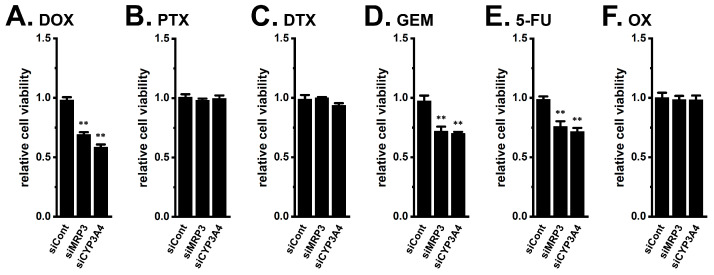
Effects of the siRNA-mediated inhibition of MRP3 and CYP3A4 on the resistance to DOX, PTX, DTX, GEM, 5-FU, and OX in the 3D spheroids of YMB-1 cells. (**A**–**F**): Effects of 1 μM DOX (**A**), 100 nM PTX (**B**), 100 nM DTX (**C**), 10 μM GEM (**D**), 10 μM 5-FU (**E**), and 1 μM OX (**F**) for 48 h on the viability of 3D spheroids transfected with siCont, siMRP3, and siCYP3A4 using the WST-1 assay (*n* = 5). Cell viability in the siCont group is expressed as 1.0. **: *p* < 0.01 vs. siCont.

To verify the functional role of MRP3 and CYP3A4, siRNA-mediated inhibition experiments were performed in YMB-1 3D spheroids (Supplementary Figures S4C,D and S5C,D). MRP3 inhibition significantly restored sensitivity to DOX, GEM, and 5-FU (*n* = 5, *p* < 0.01) but not to PTX, DTX, or OX (*n* = 5, *p* > 0.05) (Figure 6). Similarly, CYP3A4 inhibition restored DOX, GEM, and 5-FU sensitivity (*n* = 5, *p* < 0.01) (Figure 6). Cell viability remained unchanged by siRNA transfection (*n* = 5, *p* > 0.05) (Supplementary Figure S4E). Pharmacological inhibition with MK571 (20 μM, a pan-MRP inhibitor) or ketoconazole (KCZ, 0.1 μM, a potent CYP3A4 inhibitor) also reversed resistance to DOX, GEM, and 5-FU (*n* = 5, *p* < 0.01) (Supplementary Figure S8).

### 2.5. Involvement of the NRF2–CEBP Transcriptional Axis in LRRC8A Inhibition-Induced Downregulation of MRP3 and CYP3A4 in YMB-1 3D Spheroids

**Figure 7:**
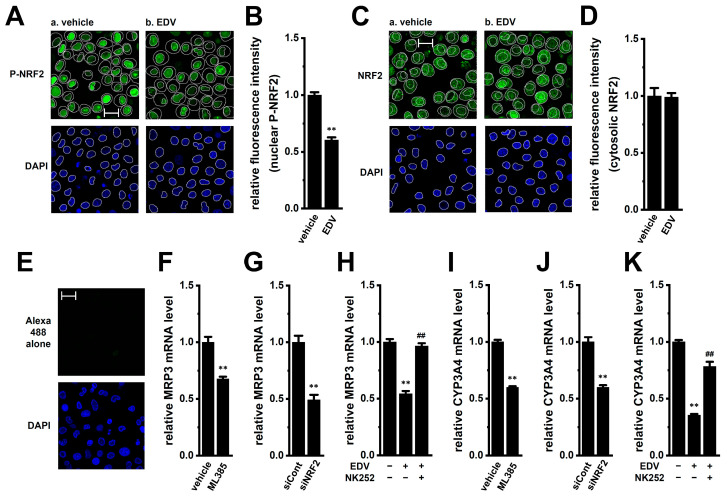
Effects of LRRC8A inhibition on the nuclear translocation of P-NRF2 in isolated cells from 3D spheroids of YMB-1 cells and effects of NRF2 inhibition on the expression levels of MRP3 and CYP3A4 transcripts in 3D spheroids. (**A**): Confocal fluorescent images of Alexa Fluor 488-labeled P-NRF2 (green) in vehicle- (a) and EDV (10 μM) (b)-treated cells isolated from YMB-1 spheroids for 2 h. Nuclear morphologies were shown by DAPI images (blue). Dashed lines show the plasma membrane and nuclear boundary. (**B**): Summarized results of the mean fluorescence intensities of Alexa Fluor 488 in nuclei (*n* = 6). In each batch (*n* = 1), more than 20 cells treated with vehicle and EDV were observed by confocal laser scanning microscopy. (**C**,**D**): Confocal fluorescent images of Alexa Fluor 488-labeled total NRF2 in vehicle- (a) and EDV (10 μM) (b)-treated cells isolated from YMB-1 spheroids for 2 h. (**E**): Confocal fluorescent images of Alexa Fluor 488 alone in untreated cells isolated from YMB-1 spheroids. (**F**,**G**,**I**,**J**): Real-time PCR examination of MRP3 (**F**,**G**) and CYP3A4 (**I**,**J**) in YMB-1 spheroids treated with vehicle and the NRF2 inhibitor, ML385 (5 μM), for 12 h (**F**,**I**) and transfected with siCont and human NRF2 siRNA (siNRF2) (**G**,**J**). After normalization to ACTB mRNA expression levels, MRP3 and CYP3A4 mRNA expression levels in the vehicle-treated and siCont-transfected groups were expressed as 1.0 (*n* = 4). (**H**,**K**): Real-time PCR examination of MRP3 (**H**) and CYP3A4 (**K**) in 3D spheroids treated (+) or untreated (−) with 10 μM EDV and 100 μM NK252, the NRF2 activator, for 12 h (*n* = 4). After normalization to ACTB mRNA expression levels, MRP3 and CYP3A4 mRNA expression levels in the vehicle control (−/−) were expressed as 1.0. The scale bar shows 20 μm. **: *p* < 0.01 vs. the vehicle control, siCont, and −/−; ^##^: *p* < 0.01 vs. +/−.

MRP3 and CYP3A4, which contribute to the reduction in anticancer drug accumulation in cancer cells, are known as NRF2 targets [31,32]. The anti-phospho-NRF2 (P-NRF2) and anti-NRF2 antibodies were labeled with the Alexa Fluor 488-conjugated secondary antibody, and the nuclei were labeled with DAPI. In isolated cells from YMB-1 3D spheroids, confocal microscopy revealed reduced nuclear P-NRF2 signals following EDV (10 μM for 2 h) treatment (*n* = 6, *p* < 0.01) (Figure 7A,B), while cytosolic NRF2 remained unchanged (Figure 7C,D). Control staining with the secondary antibody alone showed negligible background under the same imaging conditions (Figure 7E). Pharmacological blockade of NRF2 with ML385 (5 μM) (IC_50_ = approx. 2 μM) or siRNA-mediated inhibition of NRF2 (siNRF2) (Supplementary Figures S4F and S5E) significantly suppressed MRP3 and CYP3A4 gene expression (Figure 7F,G,I,J). Co-treatment with the NRF2 activator NK252 (100 μM) reversed EDV-induced downregulation (Figure 7H,K). Cell viability remained unchanged by treatment with NRF2 modulators (*n* = 5, *p* > 0.05) (Supplementary Figure S9A).

**Figure 8:**
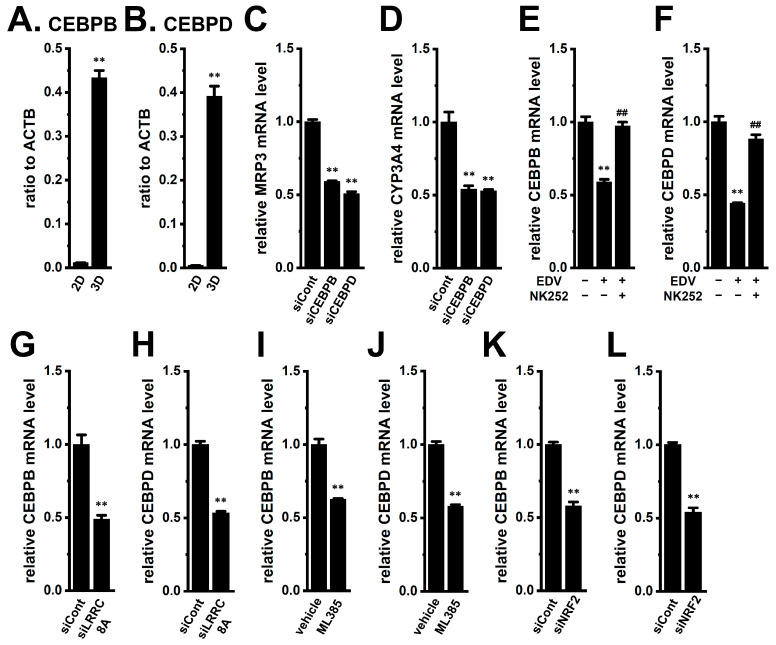
Comparison of CEBP expression between 2D monolayers and 3D spheroids of YMB-1 cells, effects of siRNA-mediated inhibition of CEBPs on the expression of MRP3 and CYP3A4 transcripts in 3D spheroids, and effects of the siRNA-mediated and pharmacological inhibition of LRRC8A and NRF2 on the expression of CEBP transcripts in 3D spheroids. (**A**,**B**): Real-time PCR examination of CEBPB (**A**) and CEBPD (**B**) in 2D monolayers and 3D spheroids. Expression levels are shown as a ratio to ACTB (*n* = 4). (**C**,**D**): Real-time PCR examination of MRP3 (**C**) and CYP3A4 (**D**) in 3D spheroids transfected with siCont, CEBPB siRNA (siCEBPB), and CEBPD siRNA (siCEBPD). After normalization to ACTB mRNA expression levels, MRP3 and CYP3A4 mRNA expression levels in the siCont are expressed as 1.0 (*n* = 4). (**E**,**F**): Real-time PCR examination of CEBPB (**E**) and CEBPD (**F**) in 3D spheroids treated (+) or untreated (−) with 10 μM EDV and 100 μM NK252 for 12 h (*n* = 4). (**G**,**H**): Real-time PCR examination of CEBPB (**G**) and CEBPD (**H**) in 3D spheroids transfected with siCont and siLRRC8A (*n* = 4). (**I**–**L**): Real-time PCR examination of CEBPB (**I**,**K**) and CEBPD (**J**,**L**) in 3D spheroids treated with vehicle and 10 μM ML385 and transfected with siCont and siNRF2. After normalization to ACTB mRNA expression levels, CEBPB and CEBPD mRNA expression levels in the vehicle control and siCont are expressed as 1.0 (*n* = 4). **: *p* < 0.01 vs. siCont and vehicle control (−/−); ^##^: *p* < 0.01 vs. +/−.

CCAAT/enhancer-binding proteins (C/EBPs, CEBPs) family transcription factors [C/EBPα (CEBPA), C/EBPβ (CEBPB), C/EBPγ (CEBPG), C/EBPδ (CEBPD), C/EBPε (CEBPE), and C/EBPζ (CEBPZ)] regulate cancer progression [25]; CEBPB and CEBPD promote cancer stemness [33,34] and act downstream of NRF2 in cancer cells [35,36]. Both CEBPB and CEBPD were markedly upregulated in YMB-1 3D spheroids (*n* = 4, *p* < 0.01) (Figure 8A,B), and their siRNA-mediated inhibition (Supplementary Figures S4G,H and S5F,G) significantly reduced MRP3 and CYP3A4 expression (*n* = 4, *p* < 0.01) (Figure 8C,D). EDV and siLRRC8A suppressed, while NK252 restored, CEBPB and CEBPD expression (Figure 8E–H). Similar suppression occurred with NRF2 inhibition (*n* = 4, *p* > 0.01) (Figure 8I–L), but not with ANO1 blockade (*n* = 4, *p* > 0.05) (Supplementary Figure S7E–H).

### 2.6. No Involvement of NADPH Oxidases (NOXs) in MRP3 and CYP3A4 Regulation in YMB-1 3D Spheroids

**Figure 9:**
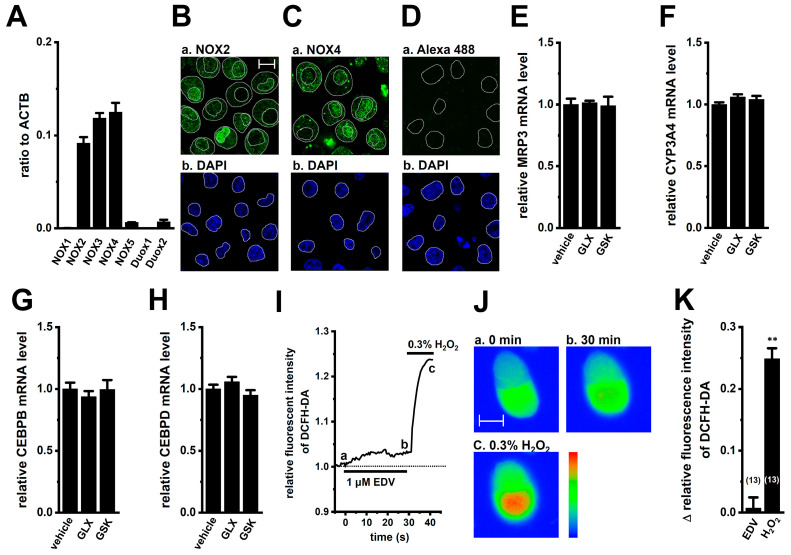
NOX family isoform expression in 3D spheroids of YMB-1 cells, effects of the pharmacological inhibition of NOX2 and NOX4 on the expression of MRP3 and CYP3A4 transcripts, and effects of treatment with EDV on ROS activity in 3D spheroids. (**A**): Real-time PCR examination of NOX1-5, Duox1 and 2 in 3D spheroids. Expression levels are shown as a ratio to ACTB (*n* = 4). (**B**,**C**): Confocal fluorescent images of Alexa Fluor 488-labeled NOX2 (**B**) and NOX4 (**C**) (green) in isolated cells from 3D spheroids. Nuclear morphologies were shown by DAPI images (blue). Dashed lines show the plasma membrane and nuclear boundary. (**D**): Confocal fluorescent images of Alexa Fluor 488 alone in untreated cells isolated from YMB-1 spheroids. (**E**–**H**): Real-time PCR examination of MRP3 (**E**), CYP3A4 (**F**), CEBPB (**G**), and CEBPD (**H**) in 3D spheroids treated with the NOX4 inhibitor, GLX351322 (10 μM), or the NOX2 inhibitor GSK2795039 (10 μM) for 12 h. MRP3, CYP3A4, CEBPB, and CEBPD mRNA expression levels in the vehicle control are expressed as 1.0. (**I**–**K**): Measurement of changes in ROS activity following the application of 1 μM EDV. The relative time courses of changes in fluorescence intensities of DCFH-DA (1.0 at time 0 s) from an isolated cell are shown (**I**). Typical pseudo-colored images of the fluorescent compound 2,7-dichlorofluorescein oxidized by ROS at 0 (a) and 30 (b) min after the treatments with 1 μM EDV in isolated cells from 3D spheroids (**J**). An amount of 0.3% H_2_O_2_ was applied at the end of the experiments (c) (**J**). The color bar is scaled in the same range, and the warmer colors represent higher ROS levels. Summarized results of changes in relative fluorescence intensity (1.0 at time 0 min) were obtained from the optical density of 2,7-dichlorofluorescein (**K**). Numbers used for experiments are shown in parentheses. The scale bar shows 10 μm. **: *p* < 0.01 vs. time 0 (1.0).

We previously showed that LRRC8A inhibition suppresses NOX2–reactive oxygen species (ROS)–NRF2 signaling in M_2_ macrophages [26]. In M_2_ macrophages, NOX2 was localized along the plasma membrane, and inhibition of NOX2 downregulated downstream cytokines of NRF2 [26]. LRRC8A functionally modulates the NOX activity, including NOX2 and NOX4 [26,37,38]. In YMB-1 3D spheroids, NOX2 and NOX4 were predominantly expressed (Figure 9A). In isolated cells from 3D spheroids, strong signals for NOX2 and NOX4 were observed in both the nucleus and perinuclear region, with very low signals along the plasma membrane (Figure 9B,C). Control staining with the secondary antibody alone showed negligible background under the same imaging conditions (Figure 9D). NOX inhibition with GLX351322 (10 μM) (IC_50_ = approx. 5 μM for NOX4) or GSK2795039 (10 μM) (IC_50_ = approx. 1 μM for NOX2) did not alter MRP3, CYP3A4, CEBPB, and CEBPD levels (Figure 9E–H). Cell viability was unchanged by treatment with them (*n* = 5, *p* > 0.05) (Supplementary Figure S9A). ROS assay showed no significant change in intracellular ROS level after EDV treatment (1 μM, 30 min; *p* > 0.05 vs. 0 min), whereas H_2_O_2_ (0.03%) robustly increased ROS (Figure 9I–K). Thus, LRRC8A does not regulate these genes through NOX–ROS signaling in YMB-1 3D spheroids.

### 2.7. Intracellular Signaling Pathways Mediating LRRC8A Inhibition-Induced NRF2 Inactivation in YMB-1 3D Spheroids

**Figure 10:**
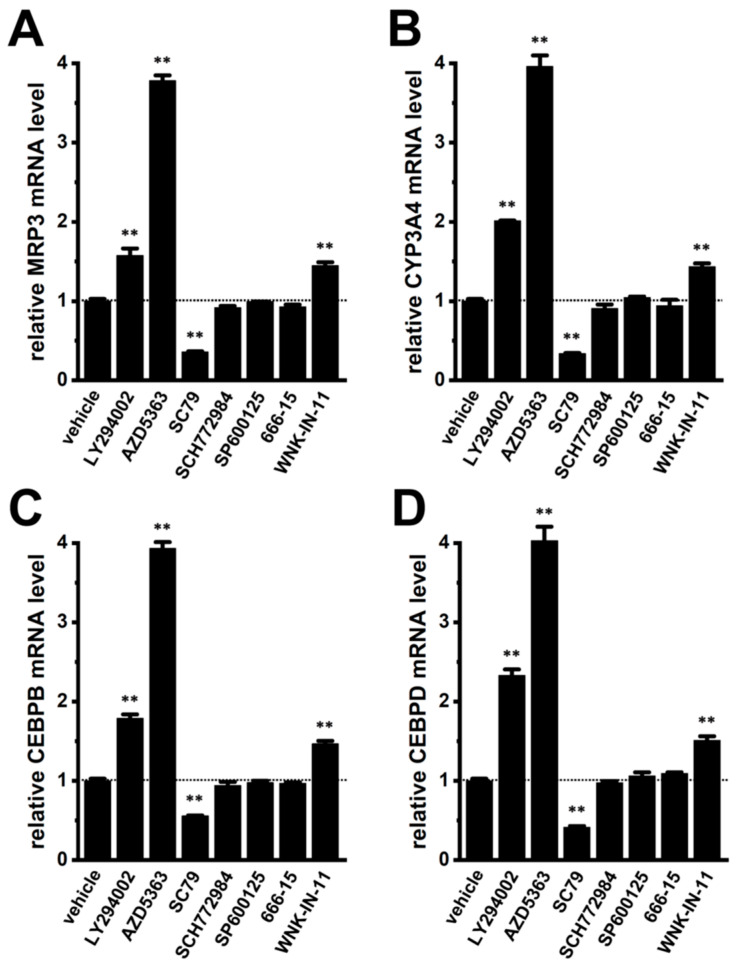
Effects of the treatment with various signal pathway inhibitors and activators on the expression of MRP3, CYP3A4, CEBPB, and CEBPD transcripts in 3D spheroids of YMB-1 cells. (**A**–**D**): Real-time PCR examination of MRP3 (**A**), CYP3A4 (**B**), CEBPB (**C**), and CEBPD (**D**) expression in vehicle-; a PI3K inhibitor, LY294002 (10 μM)-; an AKT inhibitor, AZD5363 (2 μM)-; an AKT activator, SC79 (10 μM)-; an ERK inhibitor, SCH772984 (1 μM)-; a JNK inhibitor, SP600125 (1 μM)-; a pan-CREB inhibitor, 666-15 (1 μM)-; and a WNK1 inhibitor, WNK-IN-11 (1 μM)-treated 3D spheroids for 12 h. After normalization to ACTB mRNA expression levels, MRP3, CYP3A4, CEBPB, and CEBPD mRNA expression levels in the vehicle control are expressed as 1.0 (*n* = 4). **: *p* < 0.01 vs. the vehicle control.

**Figure 11:**
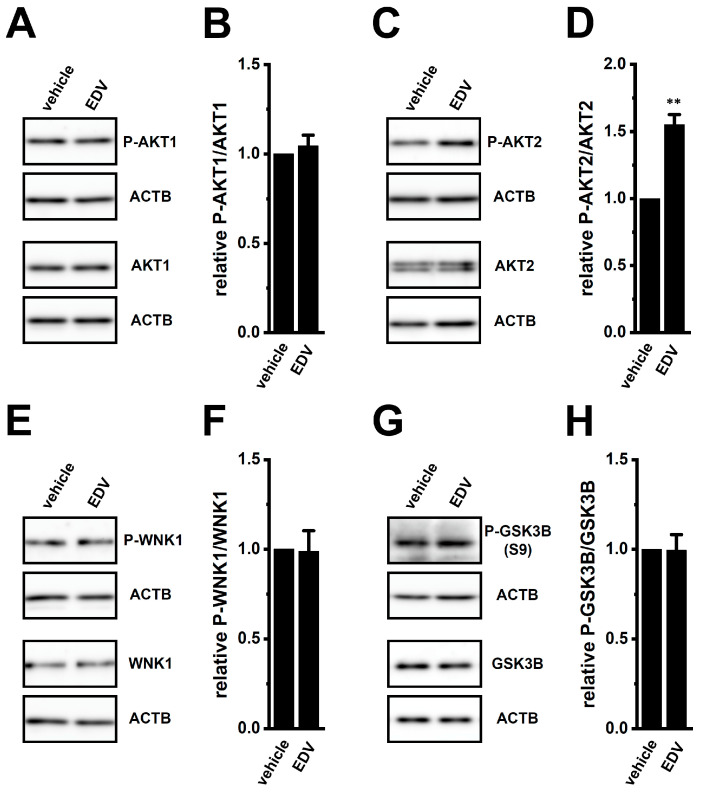
Changes in the phosphorylation levels of AKT1/2, WNK1, and GSK3B by LRRC8A inhibition in 3D spheroids of YMB-1 cells. (**A**,**C**,**E**,**G**): Protein expression of phosphorylated AKT1 (P-AKT1) and total AKT1 (AKT1) (**A**), phosphorylated AKT2 (P-AKT2) and total AKT2 (AKT2) (**C**), phosphorylated WNK1 (P-WNK1) and total WNK1 (WNK1) (**E**), and phosphorylated GSK3B (S9) (P-GSK3B) and total GSK3B (GSK3B) (**G**) in protein lysates of vehicle- and EDV (10 μM)-treated YMB-1 3D spheroids for 2 h. Blots were probed with anti-P-AKT1/AKT1, anti-P-AKT2/AKT2, anti-P-WNK1/WNK1, anti-P-GSK3B/GSK3B, and anti-ACTB antibodies. (**B**,**D**,**F**,**H**): Summarized results of the relative expression of P-AKT1/AKT1 (**B**), P-AKT2/AKT2 (**D**), P-WNK1/WNK1 (**F**), and P-GSK3B/GSK3B (**H**) were obtained from their optical densities after compensating with ACTB ones (*n* = 4). **: *p* < 0.01 vs. the vehicle control.

LRRC8A modulates multiple signaling cascades, including PI3K/AKT and JNK pathways [6,39] that play an important role in chemoresistance in many cancers, including breast cancer. Treatment with PI3K inhibitor LY294002 (10 μM) (IC_50_ = approx. 0.5 μM) or AKT inhibitor AZD5363 (2 μM) (IC_50_ = approx. 10 nM) for 12 h upregulated MRP3, CYP3A4, CEBPB, and CEBPD (*n* = 4, *p* < 0.01), whereas AKT activator SC79 (10 μM) downregulated them (*n* = 4, *p* < 0.01) in YMB-1 3D spheroids (Figure 10). In contrast, no significant changes were found in ERK, JNK, and CREB inhibition with SCH772984 (1 μM) (IC_50_ = approx. 10 nM), SP600125 (1 μM) (IC_50_ = approx. 0.1 μM), and 666-15 (1 μM) (IC_50_ = approx. 0.1 μM), respectively (Figure 10). In addition, inhibition of with-no-K (lysine) protein kinase 1 (WNK1) with WNK-IN-11(1 μM) (IC_50_ = approx. 10 nM) upregulated them (*n* = 4, *p* < 0.01) (Figure 10). NRF2 activity is enhanced by AKT-mediated phosphorylation through WNK1 [40,41] and glycogen synthase kinase 3β (GSK3B) [42]. We therefore examined the LRRC8A–AKT interplay. EDV treatment (10 μM, 2 h) significantly increased AKT2 phosphorylation (*n* = 4, *p* < 0.01) without altering AKT1, WNK1, or GSK3B (*n* = 4, *p* > 0.05) (Figure 11). Cell viability was unchanged by treatment with the compounds used in this section (*n* = 5, *p* > 0.05) (Supplementary Figure S9B). Thus, LRRRC8A inhibition likely downregulates the NRF2–CEBPB/D axis through AKT2 activation in YMB-1 3D spheroids. Consistently, AKT blockade with AZD5363 increased nuclear P-NRF2 signals without affecting P-WNK1 distribution (Supplementary Figure S10).

### 2.8. Epigenetic Modification of MRP3 and CYP3A4 by MicroRNAs in YMB-1 3D Spheroids

**Figure 12:**
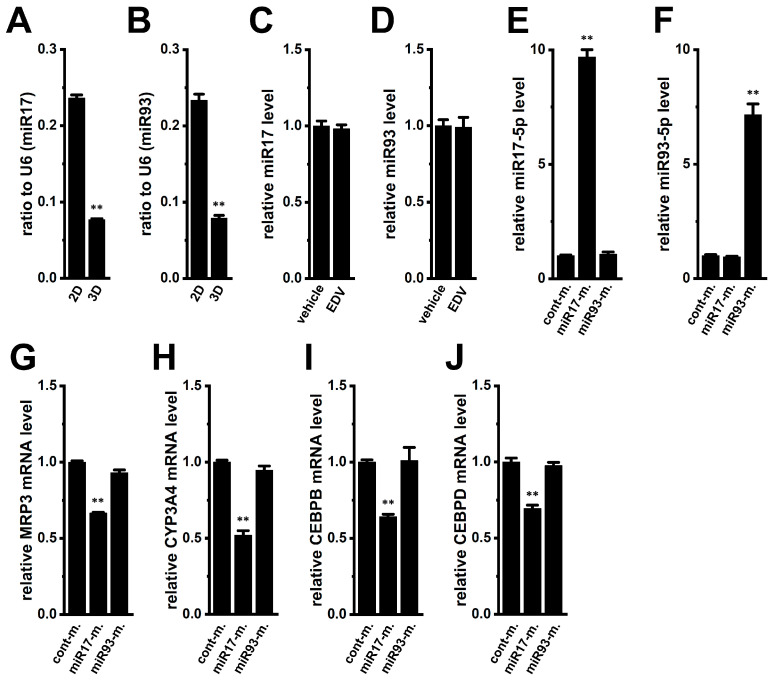
Comparison of expression levels of miR17 and miR93 between 2D monolayers and 3D spheroids of YMB-1 cells, effects of the pharmacological inhibition of VRAC with 10 μM EDV for 12 h on expression levels of miR17 and miR93, and effects of miR17-5p and miR93-5p mimics on the expression levels of MRP3 and CYP3A4 transcripts in the 3D spheroids. (**A**,**B**): Real-time PCR of miR17 (**A**) and miR93 (**B**) in 2D monolayers and 3D spheroids (*n* = 4). Expression levels are shown as a ratio to U6. (**C**,**D**): Real-time PCR of miR17 (**C**) and miR93 (**D**) in the vehicle- and EDV (10 μM) -treated 3D spheroids for 12 h (*n* = 4). (**E**,**F**): Real-time PCR of miR17-5p (**E**) and miR93-5p (**F**) in 3D spheroids transfected with control miR mimic (cont-m.), miR17-5p-mimic (miR17-m.), and miR93-5p-mimic (miR93-m.) (*n* = 4). After normalization to U6 expression levels, miR17-5p and miR93-5p expression levels in cont-m. were expressed as 1.0. (**G**–**J**): Real-time PCR of MRP3, CYP3A4, CEBPB, and CEBPD in 3D spheroids transfected with cont-m., miR17-m., and miR93-m. (*n* = 4). After normalization to ACTB mRNA expression levels, MRP3 (**G**), CYP3A4 (**H**), CEBPB (**I**), and CEBPD (**J**) mRNA expression levels in cont-m. were expressed as 1.0. **: *p* < 0.01 vs. 2D and cont-m.

Aberrant microRNA (miRNA) expression affects cancer stemness and therapy resistance, and a high-throughput miRNA screening system has been developed using 3D cancer spheroid models [43]. Overexpression of miR17 and miR93 reduces stemness gene expression and sensitizes breast cancer cells to chemotherapy [44]; both also regulate NRF2 [45]. miR17 and miR93 were significantly decreased during spheroid formation (*n* = 4, *p* < 0.01) (Figure 12A,B), and were unaffected by EDV treatment (*n* = 4, *p* > 0.05) (Figure 12C,D). Transfection with miR-17-5p mimics, but not miR-93-5p, reduced MRP3 and CYP3A4 expression (*n* = 4, *p* < 0.01) (Figure 12G,H) and concomitantly decreased CEBPB/D levels (Figure 12I,J). The miR-17-5p and miR-93-5p mimics increased their respective expression levels by approximately 10-fold and 7-fold, respectively (Figure 12E,F). Thus, reduced miR-17 may contribute to MRP3/CYP3A4 upregulation during spheroid formation (cancer stemness) through an LRRC8A-independent epigenetic mechanism.

### 2.9. Comparable Studies in 3D Spheroids of MDA-MB-468 Cells

**Figure 13:**
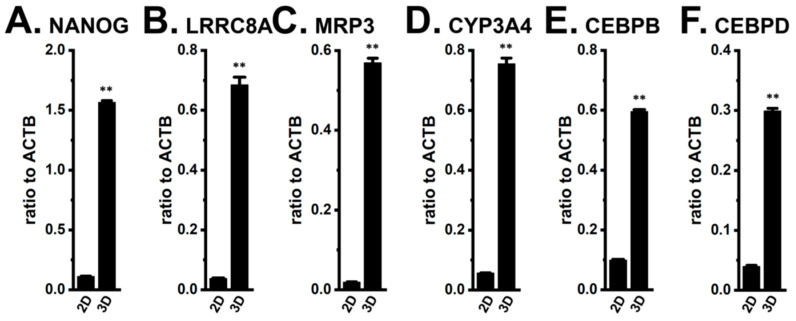
Comparison of the expression levels of NANOG, LRRC8A, MRP3, CYP3A4, CEBPB, and CEBPD transcripts between 2D monolayers and 3D spheroids of human breast cancer MDA-MB-468 cells. (**A**–**F**): Real-time PCR examination of the NANOG (**A**), LRRC8A (**B**), MRP3 (**C**), CYP3A4 (**D**), CEBPB (**E**), and CEBPD (**F**) transcripts in ‘2D’ and ‘3D’ groups. Expression levels are shown as a ratio to ACTB (*n* = 4). **: *p* < 0.01 vs. ‘2D’.

As shown in Figure 13A,B, NANOG and LRRC8A were significantly overexpressed in MDA-MB-468 3D spheroids compared with its 2D monolayers (*n* = 4, *p* < 0.01). Consistently, the transcript levels of MRP3, CYP3A4, CEBPB, and CEBPD markedly increased during spheroid formation (*n* = 4, *p* < 0.01) (Figure 13C–F).

**Figure 14:**
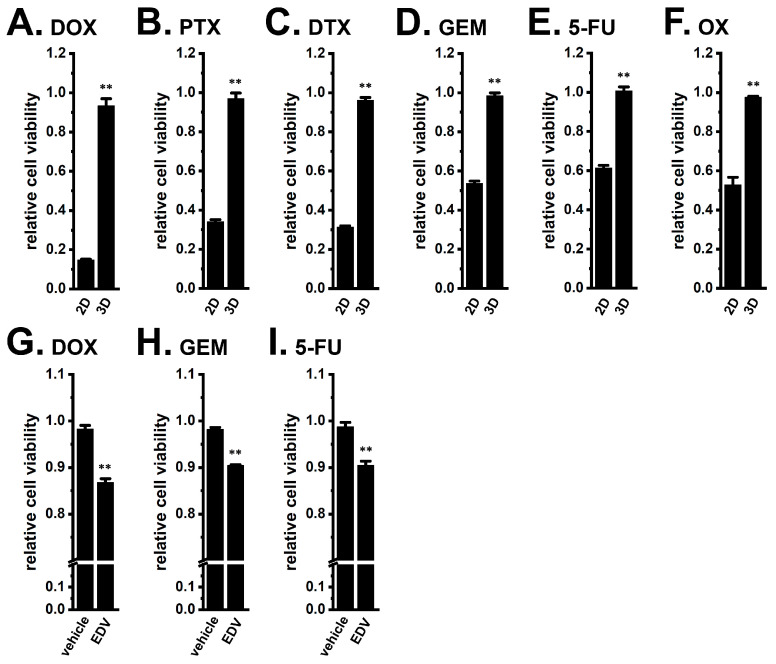
Effects of chemotherapeutic agents on the viability of 2D monolayers and 3D spheroids of MDA-MB-468 cells, and effects of LRRC8A inhibition on resistance to them in MDA-MB-468 3D spheroids. (**A**–**F**): Effects of the treatment with 1 μM DOX (**A**), 100 nM PTX (**B**), 100 nM DTX (**C**), 10 μM GEM (**D**), 10 μM 5-FU (**E**), and 1 μM OX (**F**) for 48 h in ‘2D’ and ‘3D’ using the WST-1 assay (*n* = 5). (**G**–**I**): Effects of the treatment with DOX (**G**), GEM (**H**), and 5-FU (**I**) for 48 h on the cell viability of MDA-MB-468 3D spheroids pre-treated with vehicle and 10 μM EDV for 24 h (*n* = 5). Cell viability in the untreated group was expressed as 1.0. **: *p* < 0.01 vs. ‘2D’.

Resistance to chemotherapeutic agents, DOX (1 μM), PTX (100 nM), DTX (100 nM), GEM (10 μM), 5-FU (10 μM), and OX (1 μM), was observed in MDA-MB-468 3D spheroids (Figure 14A–F). Concentration–response profiles for all agents in 2D and 3D cultures are summarized in Supplementary Figure S11. Notably, pre-treatment of MDA-MB-468 3D spheroids with EDV (10 μM) for 24 h significantly reversed resistance to DOX, GEM, and 5-FU (*n* = 5, *p* < 0.01) (Figure 14G–I).

**Figure 15:**
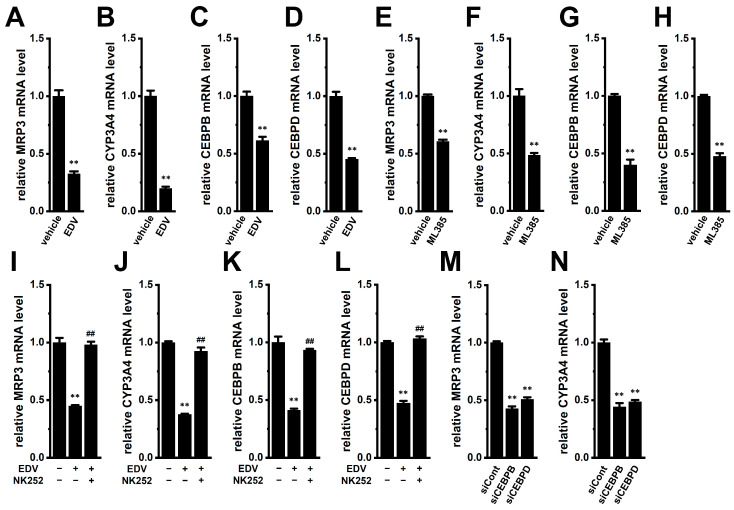
Effects of the pharmacological modulation of LRRC8A and NRF2 on the expression levels of MRP3, CYP3A4, CEBPB, and CEBPD and effects of the siRNA-mediated inhibition of CEBPB and CEBPD on the expression levels of MRP3 and CYP3A4 in MDA-MB-468 3D spheroids. (**A**–**D**): Real-time PCR examination of MRP3 (**A**), CYP3A4 (**B**), CEBPB (**C**), and MRP5 (**D**) in 3D spheroids treated with vehicle and 10 μM EDV for 12 h. (**E**–**H**): Real-time PCR examination of MRP3 (**E**), CYP3A4 (**F**), CEBPB (**G**), and CEBPD (**H**) in MDA-MB-468 3D spheroids treated with vehicle and 5 μM ML385 for 12 h. (**I**–**L**): Real-time PCR examination of MRP3 (**I**), CYP3A4 (**J**), CEBPB (**K**), and CEBPD (**L**) in 3D spheroids treated (+) or untreated (−) with 10 μM EDV and 100 μM NK252 for 12 h (*n* = 4). (**M**,**N**): Real-time PCR examination of MRP3 (**M**) and CYP3A4 (**N**) in 3D spheroids transfected with siCont, siCEBPB, and siCEBPD. After normalization to ACTB mRNA expression levels, MRP3, CYP3A4, CEBPB, and CEBPD mRNA expression levels in the vehicle control (−/−) and siCont were expressed as 1.0. **: *p* < 0.01 vs. the vehicle control (−/−) and siCont; ^##^: *p* <0.01 vs. +/−.

Similar to the findings in YMB-1 3D spheroids, the treatment with EDV for 12 h significantly reduced the transcript levels of MRP3, CYP3A4, CEBPB, and CEBPD in MDA-MB-468 3D spheroids (*n* = 4, *p* < 0.01) (Figure 15A–D). Consistently, siRNA-mediated inhibition of LRRC8A also significantly downregulated these genes (*n* = 4, *p* < 0.01) (Supplementary Figure S12A,D–G). In addition, pharmacological inhibition of NRF2 with ML385 (5 μM) significantly reduced their expression (Figure 15E–H), whereas co-treatment with the NRF2 activator NK252 (100 μM) reversed EDV-induced downregulation in MDA-MB-468 3D spheroids (Figure 15I–L). Furthermore, siRNA-mediated inhibition of CEBPB and CEBPD also significantly decreased the expression of these downstream targets, MRP3 and CYP3A4 (*n* = 4, *p* < 0.01) (Figure 15M,N).

**Figure 16:**
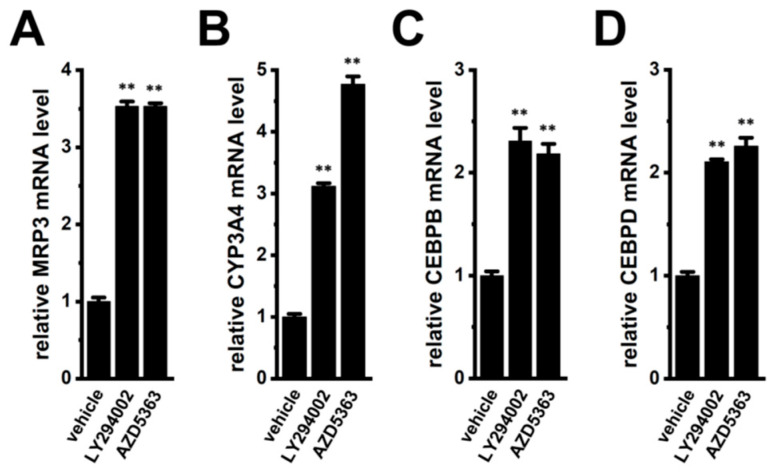
Effects of the treatment with LY294002 and AZD5363 on the expression of MRP3, CYP3A4, CEBPB, and CEBPD transcripts in MDA-MB-468 3D spheroids. (**A**–**D**): Real-time PCR examination of MRP3 (**A**), CYP3A4 (**B**), CEBPB (**C**), and CEBPD (**D**) expression in vehicle-, LY294002 (10 μM)-, and AZD5363 (2 μM)-treated 3D spheroids for 12 h. After normalization to ACTB mRNA expression levels, MRP3, CYP3A4, CEBPB, and CEBPD mRNA expression levels in the vehicle control are expressed as 1.0 (*n* = 4). **: *p* < 0.01 vs. the vehicle control.

As shown in Figure 10, PI3K and AKT inhibitors upregulated MRP3, CYP3A4, CEBPB, and CEBPD in YMB-1 3D spheroids. Similarly, treatment of MDA-MB-468 3D spheroids with LY294002 (10 μM) or AZD5363 (2 μM) for 12 h significantly increased the expression levels of these genes (*n* = 4, *p* < 0.01) (Figure 16).

## 3. Discussion

In the present study, we identified that LRRC8A is a pivotal regulator of chemoresistance in a breast cancer 3D spheroid model that recapitulates key characteristics of cancer stemness. Our findings demonstrate that LRRC8A is highly expressed in 3D spheroids and functions as a critical determinant of multidrug resistance through modulation of the NRF2-CEBPB/D transcriptional axis (Figure 1, Figure 2, Figure 3, Figure 7, Figure 8, Figure 13, Figure 14 and Figure 15). Pharmacological and genetic inhibition of LRRC8A restored sensitivity to DOX, GEM, and 5-FU (Figure 3 and Figure 14), suggesting a selective reliance of nucleoside- and anthracycline-based therapies on LRRC8A-mediated signaling. On the other hand, sensitivity to taxanes and platinum agents was not affected by LRRC8A inhibition (Figure 3), indicating that LRRC8A regulates specific exclusion and metabolism pathways rather than inducing global cytotoxicity. DOX, GEM, and 5-FU represent mechanistically distinct classes, including DNA intercalation and topoisomerase II inhibition for DOX and nucleoside analog-mediated inhibition of DNA synthesis for GEM and 5-FU. Despite their distinct mechanisms of action, inhibition of LRRC8A consistently restored sensitivity to all three agents. These findings suggest that LRRC8A-mediated transcriptional regulation of drug efflux and metabolic pathways operates upstream of drug-specific cytotoxic mechanisms, thereby functioning as a common regulatory node in chemoresistance.

As shown in Figure 4, Figure 5 and Figure 13, MRP3 and CYP3A4 were markedly upregulated during spheroid formation. MRP3, a well-established downstream molecule of NRF2 in cancer [46,47], is implicated in drug resistance and stemness in breast cancer [48]. Downregulation of MRP3 renders cancer cells increasingly susceptible to DOX in xenograft mouse models in vivo, suggesting a critical role of MRP3 in mediating chemoresistance in breast cancer [48]. CYP3A4, likewise a downstream effector of NRF2 and overexpressed in malignant breast cancer [40,49], contributes to chemoresistance and stem-like traits [50]. A strong correlation between CYP3A4 levels and DOX sensitivity has been observed in 3D spheroids and clinical contexts [50,51]. Our study presents the significant findings that regulation of MRP3 and CYP3A4 expression in the tumor microenvironment provides a physiologically relevant platform for investigating mechanisms of resistance to DOX, GEM, and 5-FU.

Mechanistically, LRRC8A inhibition led to significant downregulation of MRP3 and CYP3A4, two major determinants of drug export and metabolism (Figure 4, Figure 5 and Figure 15). Both genes were transcriptionally governed by the NRF2–CEBPB/CEBPD axis, as indicated by the decrease in nuclear phosphorylated NRF2 without changes in cytosolic NRF2 levels and suppression of its downstream transcription factors following LRRC8A inhibition (Figure 7 and Figure 8). Rescue experiments with the NRF2 activator NK252 restored MRP3 and CYP3A4 expression, further supporting the central role of NRF2 signaling in LRRC8A-mediated chemoresistance. CEBPB and CEBPD, which promote stemness and chemoresistance in various cancers, including breast cancer [52,53], were similarly repressed by LRRC8A inhibition (Figure 8 and Figure 15). Unlike M_2_ macrophages, where LRRC8A modulates NOX2-dependent ROS signaling [26], LRRC8A-regulated chemoresistance in YMB-1 3D spheroids occurred independently of NOX-ROS pathways despite increased NOX2 and NOX4 expression (Figure 9).

A notable finding of this study is the selective involvement of AKT2 in LRRC8A-mediated NRF2 regulation (Figure 10, Figure 11 and Figure 16). The increased phosphorylation of AKT2 following LRRC8A inhibition (Figure 11), together with the reciprocal activation of NRF2 upon pharmacological inhibition of AKT (Supplementary Figure S10), positions AKT2 as an unexpected suppressor of NRF2 signaling in this context. AKT2, a major effector of the PI3K–AKT–NRF2 signaling axis, is known to contribute to chemoresistance in breast cancer stem cells [54], and activation of the PI3K/AKT pathway promotes chemoresistance and stemness-like phenotypes in breast cancer cells [55]. However, in the present study, inhibition of either PI3K or AKT upregulated MRP3 and CYP3A4 expression in 3D spheroids via NRF2 activation (Figure 10A,B, Supplementary Figure S10A,B). Consistent with these findings, a recent study demonstrated that AKT inhibition enhances NRF2 activity by promoting nuclear translocation of NRF2 in stimulated macrophage cells [56], suggesting that AKT signaling can prevent nuclear accumulation of NRF2. AKT2 silencing has been shown to increase ALDH1A1 expression in breast cancer cell lines, including YMB-1 cells [54], while NRF2 knockdown decreases it [57]. These reports support the notion that AKT2 activation can suppress NRF2 signaling. The precise molecular mechanism by which LRRC8A modulates AKT2 activity remains unclear; however, the present results raise the possibility that the LRRC8A–AKT2 axis creates a unique signaling hub controlling NRF2-mediated transcriptional programs in stemness-enriched breast cancer cells.

It has been reported that LRRC8A-related VRACs directly transport platinum-containing anticancer drugs, cisplatin and carboplatin [58,59]. It remains to be determined whether DOX, GEM, 5-FU, or their intracellular metabolites can function as substrates of VRACs. Future studies should therefore investigate whether these compounds are directly transported via VRACs, which could be assessed by acutely quantifying their accumulation in 3D spheroids following pharmacological inhibition of LRRC8A.

RNA modifications play crucial roles in various RNA metabolic processes, including stability, processing, and translation, particularly in cancer cells [60]. In parallel, epigenetic mechanisms, such as DNA methylation, histone modification, and non-coding RNA regulation, contribute to cancer initiation and progression [61]. Both RNA modification and epigenetic regulation are implicated in the development of chemoresistance, and targeting these processes has emerged as a promising strategy to overcome drug resistance [62]. miR-17-5p, an activator of the PI3K/AKT pathway through PTEN suppression [63], is also implicated in NRF2 activation associated with PTEN loss in human carcinogenesis [64]. We identified an LRRC8A-independent mechanism involving the acquisition of chemoresistance through miR-17 downregulation by spheroid formation (Figure 12). Restoration of miR-17 reduced MRP3, CYP3A4, and CEBPB/D expression, suggesting that decreased miR-17 integrates into stemness-driven reinforcement of multidrug resistance. Furthermore, the class III histone deacetylase sirtuin 1 (SIRT1) has been implicated in cancer stemness and drug resistance [65,66]. As shown in Supplementary Figure S13A–C, reduced SIRT1 expression emerged as a potential molecule contributing to the upregulation of LRRC8A during spheroid formation in YMB-1 3D spheroids, consistent with previous studies linking SIRT1 downregulation to cancer stemness and poor prognosis. Pharmacological inhibition of SIRT1 with Ex527 (1 μM) (IC_50_ = approx. 0.1 μM) significantly increased LRRC8A transcript levels in YMB-1 cells as 2D monolayers, whereas no such effect was observed in 3D spheroids (Supplementary Figure S13D,E). These findings suggest that SIRT1 inhibition may contribute, at least in part, to LRRC8A induction during spheroid formation. However, to establish a definitive epigenetic mechanism, further validation is required, including chromatin immunoprecipitation assays to assess SIRT1 binding to the LRRC8A promoter region and to evaluate associated histone modifications.

As shown in Figure 3 and Figure 6, inhibition of LRRC8A, MRP3, or CYP3A4 did not overcome resistance to taxanes (PTX and DTX) in YMB-1 3D spheroids. Recent evidence indicates that inhibition of LAT1/SLC7A5 sensitizes breast cancer cells to taxanes [67,68]. SLC7A5 transcripts were significantly upregulated in 3D spheroids (Supplementary Figure S14A); however, no significant changes were found by LRRC8A inhibition (Supplementary Figure S14B,C). Importantly, blockade of SLC7A5 with JPH203 (10 μM) (IC_50_ = approx. 5 μM) significantly restored taxane sensitivity in 3D spheroids (Supplementary Figure S14D,E). These results suggest that amino acid metabolism and glycolytic regulation contribute specifically to taxane resistance in breast cancer stem-like cells. SLC7A5 inhibition also overcame resistance to DOX and GEM but not 5-FU and OX (Supplementary Figure S14F–I).

In M_2_ macrophages, LRRC8A activates NOX2-dependent ROS pathways, and NOX2 is distributed along the plasma membrane [26]. In 3D spheroids, NOX2/4 were mainly localized in perinuclear and nuclear regions (Figure 9B,C). The integral membrane protein p22^phox^ forms a heterodimeric enzyme complex with NOXs, and reduced enzymatic activity by its downregulation results in decreased ROS generation [69]. The expression level of p22^phox^ was relatively low in YMB-1 3D spheroids (Supplementary Figure S15A), and approximately one-tenth in THP-1-differentiated M_2_ macrophages (Supplementary Figure S15D). Although LRRC8A inhibition suppresses the production of ROS and inflammatory cytokines by downregulating the expression of NOX4 and p22^phox^ in immune cells [45], in 3D spheroids, pharmacological inhibition of NOX2 and NOX4 did not affect MRP3 or CYP3A4 levels (Figure 9E,F), and LRRC8A inhibition did not alter ROS production or NOX2/4 and p22^phox^ expression (Figure 9J,K, Supplementary Figure S15B,C,E–H). These results indicate that LRRC8A-mediated chemoresistance in 3D spheroids is independent of the NOX−ROS signaling.

In the present study, we did not evaluate the significance of LRRC8A using a low-dose chronic exposure model because it was difficult to keep the long-term stability of the cancer stem cell-like phenotype. Low-dose, chronic exposure experiments are essential for understanding the clinically relevant evolution of chemoresistance. Further studies using cancer stem-like cells, which can drive long-term resistance, will be necessary to clarify the clinical significance of LRRC8A to overcoming chemoresistance in breast cancer treatment. Activation of NRF2 signaling, drug efflux transporters, metabolic rewiring, and epigenetic remodeling are often missed in short-term exposure and are induced following long-term exposure; however, in the present study, they were observed in the 3D spheroid model.

**Figure 17:**
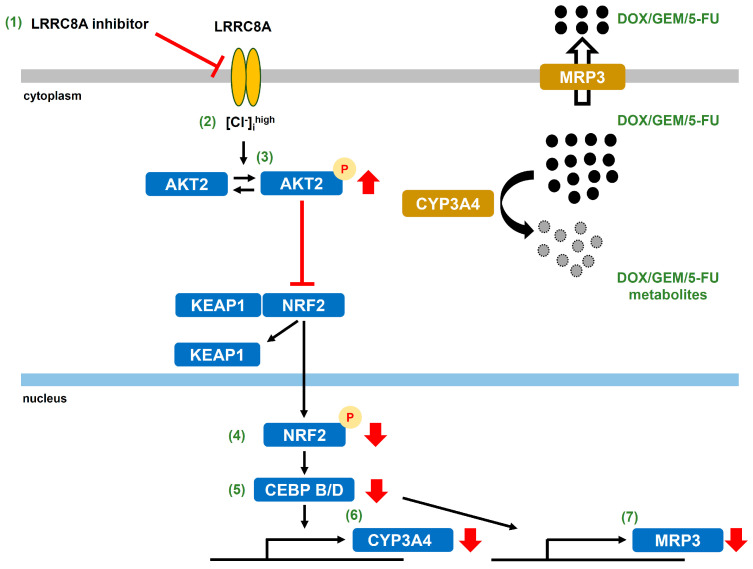
Schematic diagram of the intracellular signaling pathways involved in LRRC8A-mediated MRP3 and CYP3A4 transcription in the 3D spheroid model of human breast cancer. LRRC8A inhibition downregulates MRP3 and CYP3A4 through the NRF2–CEBP transcriptional axis.

Collectively, our results establish LRRC8A as a central regulator of drug resistance in stem-like cancer cells and provide mechanistic insights linking LRRC8A-downstream signaling to transcriptional networks governing chemoresistance (Figure 17). LRRC8A thus represents a promising therapeutic target for overcoming drug resistance in breast cancer. The combined administration of low-dose LRRC8A inhibitors with agents targeting CYP3A4-mediated metabolism or MRP3-mediated efflux may achieve safe and efficient anticancer effects, and it may offset the side effects caused by high concentrations of chemotherapeutic agents.

## 4. Materials and Methods

### 4.1. Materials and Reagents

RPMI 1640 (189-02025), Dulbecco’s Modified Eagle’s Medium (D-MEM) (043-30085) media, paclitaxel (161-28164), 0.25 w/v% Trypsin Solution with Phenol Red (201-18841), DNase-free RNase (313-01461), and doxorubicin hydrochloride (040-21521) were purchased from FUJIFILM Wako Pure Chemicals (Osaka, Japan). Fetal bovine serum (FBS) (Product code: 172012), GLX351322 (SML2546), 4′,6-diamidino-2-phenylindole (DAPI) (D2542), and Phosphatase Inhibitor Cocktails (P5726, P0044) were from Sigma-Aldrich (St. Louis, MO, USA). DiBAC_4_(3) (D545), Fura 2-AM (F025), WST-1 (W201), 1-Methoxy PMS (M003), propidium iodide (PI) (P378), and ROS assay kit (photo-oxidation-resistant DCFH-DA) (R253) were from Dojindo (Kumamoto, Japan). Docetaxel (D4102) was from TCI (Tokyo, Japan). Endovion (EDV, NS3728) (HY-105917), ANO1-IN-1 (HY-146320), SP600125 (HY-12041), NK252 (HY-19734), ketoconazole (KCZ, HY-B0105), SC79 (HY-18749), MK571 (HY-19989), hsa-miR17-5p miRNA mimic (HY-R00326), hsa-miR93-5p mimic (HY-R02520), miRNA mimic Negative Control (HY-R04602), GEM (HY-17026), 5-FU (HY-90006), OX (HY-17371), and GSK2795039 (HY-18950) were from MedChemExpress (Monmouth Junction, NJ, USA). AZD5363 (15406), GSK2795039 (33777), SCH772984 (19166), WNK-IN-11 (29676), and ionomycin (10004974) were from Cayman Chemical (Ann Arbor, MI, USA). ML385 (S8790), 666-15 (S8846), Ex527 (S1541) were from Selleckchem (Yokohama, Japan). LY294002 (CS-0016977) was from ChemScene (Monmouth Junction, NJ, USA). ULTRARIPA kit for Lipid Raft (F015) was from BioDynamics Laboratory (Tokyo, Japan). Luna Universal qPCR Master Mix (M3003E) was from New England Biolabs Japan (Tokyo, Japan). Lipofectamine^®^ RNAiMAX (13778075) and SYBR Green qPCR Master Mix (A66732) were from Thermo Fisher Scientific (Waltham, MA, USA). ReverTra Ace (TRT-101) was from ToYoBo (Osaka, Japan). Flat-bottomed dishes and plates were from Corning (Corning, NY, USA). PrimeSurface 96U plates (MS-9096U) were from Sumitomo Bakelite (Tokyo, Japan). Select Pre-designed/Validated siRNAs as a negative control (Pre-designed, No.1), LRRC8A (Pre-designed, ID#: s32108), ANO1 (Pre-designed, ID#: s30184), NRF2 (Pre-designed, ID#: s9493), MRP3 (Pre-designed, ID#: s16600), CYP3A4 (Validated, ID#: s3846), CEBPB (Pre-designed, ID#: s2892), CEBPD (Pre-designed, ID#: s2895), and WNK1 (Validated, ID#: 1174) were from Life Technology Japan (Tokyo, Japan). FastGene RNA Premium kit and FastGene miRNA Enhancer kit were from Nippongenetics (Tokyo, Japan). Mir-X miRNA First-Strand Synthesis kit was from TaKaRa (Osaka, Japan). CytoFix/Perm kit (554714) was from BD Biosciences (Franklin Lakes, NJ, USA). PCR primers were from Nihon Gene Research Laboratories (Sendai, Japan). SuperSignal West Pico PLUS Chemiluminescent Substrate (34580) was from Thermo Fisher Scientific (Waltham, MA, USA). Primary and secondary antibodies were listed in Table S2. Other chemicals and reagents were from Sigma-Aldrich and FUJIFILM Wako Pure Chemicals.

### 4.2. Cell Culture

The human breast cancer cell lines YMB-1 and MDA-MB-468 were obtained from the RIKEN Cell Bank (Osaka, Japan) and American Type Culture Collection (ATCC, Manassas, VA, USA), respectively. The cell lines were cultured in RPMI 1640 (for YMB-1) and D-MEM (for MDA-MB-468) media supplemented with 10% FBS and penicillin (100 units/mL)/streptomycin (100 μg/mL) at 37 °C in a humidified atmosphere containing 5% CO_2_. Flat-bottomed plates and dishes were used for two-dimensional (2D) monolayer cell culture. For 3D spheroid cell culture, cell suspensions were seeded onto a PrimeSurface 96U plate at 10^4^ cells/well and then cultured for 7 days.

### 4.3. RNA Extraction, cDNA Synthesis, and Real-Time PCR

Total RNA was isolated from cancer cells using the conventional acid guanidinium thiocyanate-phenol-chloroform extraction method. The concentration and quality of RNA were confirmed using the microvolume spectrophotometer, NanoDrop One (Thermo Fisher Scientific). Reverse transcription was performed using ReverTra Ace with random hexanucleotides. miRNAs were extracted using FastGene RNA Premium kit with FastGene miRNA Enhancer, and cDNAs from miRNAs were synthesized using Mir-X miRNA First-Strand Synthesis kit. Quantitative, real-time PCR was conducted using the Applied Biosystems 7500 Fast Real-Time PCR System (Thermo Fisher Scientific). PCR primers of human origin are listed in Table S1. Relative expression levels were calculated using the 2^−ΔΔCt^ method and normalized to ACTB.

### 4.4. Western Blots

Whole-cell and lipid raft lysates were extracted using the ULTRARIPA kit. Equal amounts of protein were subjected to SDS-PAGE and immunoblotting with primary antibodies, and were then incubated with secondary antibodies listed in Table S2. An enhanced chemiluminescent Western blotting detection reagent was used to detect the bound antibody. The resulting images were analyzed using Amersham Imager 600 (GE Healthcare Japan, Tokyo, Japan). The optical density of the protein band signal relative to that of the ACTB signal was calculated using ImageJ software (Ver. 1.42, NIH, Bethesda, MD, USA), and protein expression levels in the vehicle control were then expressed as 1.0.

### 4.5. Cell Viability Assay

The WST-1 assay was performed to assess the in vitro cytotoxicity of chemotherapies [29,32]. Briefly, using a density of 10^4^ cells/mL, cells were cultured in duplicate in 96-well plates for 7 days (for the 3D spheroids) and 1 day (for the 2D monolayers). Cells were then treated with the chemotherapeutic agents for 2 days. Two hours after the addition of WST-1 reagent to each well, absorbance was measured using the microplate reader SpectraMax 384 (Molecular Devices Japan, Tokyo, Japan) at a test wavelength of 450 nm and a reference wavelength of 650 nm.

### 4.6. Transfection with siRNAs and miRNA Mimics

Lipofectamine RNAiMAX reagent was used in the siRNA-mediated inhibition of target genes and the miRNA mimic-mediated activation of miR17-5p and miR93-5p, according to the manufacturer’s protocol. Silencer Select Pre-designed/Validated siRNAs and pre-designed miRNAs were transfected into adherent monolayer cells. Twenty-four hours later, the transfected cells were seeded onto PrimeSurface 96U plates. The expression levels of the target transcripts were assessed using the real-time PCR assay.

### 4.7. Measurement of the Membrane Potential and Intracellular Ca^2+^ Concentrations by Fluorescence Indicators, DiBAC_4_(3) and Fura 2-AM

The membrane potential was measured using the fluorescence voltage-sensitive dye DiBAC_4_(3) [26,29]. The cells were continuously incubated with 100 nM DiBAC_4_(3) throughout the experiments. In membrane potential imaging, cells loaded with DiBAC_4_(3) were illuminated at a wavelength of 490 nm. The intracellular Ca^2+^ concentrations were measured using the fluorescent Ca^2+^ indicator dye Fura 2-AM. Cells were incubated with 10 μM Fura 2-AM for 30 min at room temperature. Cells loaded with Fura 2 were alternatively illuminated at wavelengths of 340 and 380 nm. Fluorescence images were recorded on an ORCA-Flash2.8 digital camera (Hamamatsu Photonics, Hamamatsu, Japan). Data collection and analyses were performed using an HCImage system (Hamamatsu Photonics). Images were measured every 5 s, and the values of fluorescence intensity were determined by measuring the average for 1 min (12 images). The fluorescence intensity of Fura 2 was expressed as measured 340/380 nm fluorescence ratios after background subtraction.

### 4.8. Visualization Analysis of Cellular Distribution of P-NRF2, NRF2, NOX2, NOX4, and P-WNK1

Cells were isolated from 3D spheroids with trypsin solution and were fixed and permeabilized using the CytoFix/Perm kit. The antibodies for P-NRF2, NRF2, NOX2, NOX4, P-WNK1, and WNK1 shown in Table S2 (for immunocytochemistry, ICC) were labeled with an Alexa Fluor 488-conjugated secondary antibody. The nuclei were visualized by DAPI staining. After seeding onto the glass-bottomed dishes, fluorescence images were visualized using a confocal laser scanning microscope system (Nikon A1R, Tokyo, Japan). Image data were quantitatively analyzed using ImageJ software. For each experimental replicate (*n* = 1), fluorescence intensities were obtained from at least four frame images containing more than 20 cells. Summarized data were derived from six independently differentiated dishes. Cell immunostaining, imaging acquisition, and quantitative analyses were independently performed by separate investigators to minimize potential bias.

### 4.9. Measurement of ROS

To measure intracellular ROS levels, isolated cells with trypsin solution were seeded onto the glass-bottomed dish and then were loaded with 10 μM photo-oxidation-resistant DCFH-DA dye at 37 °C for 30 min, according to the manufacturer’s instructions. The dyes were washed out and illuminated at a wavelength of 490 nm. Fluorescence images of the fluorescent compound 2,7-dichlorofluorescein oxidized by ROS were recorded using the ORCA-Flash2.8 digital camera (Hamamatsu Photonics). Data collection and analyses were performed using the HCImage system (Hamamatsu Photonics).

### 4.10. Statistical Analyses

Statistical analyses were performed using the statistical software XLSTAT (version 2013.1). To assess the significance of differences between two groups and among multiple groups, unpaired/paired Student’s *t*-tests with Welch’s correction or Tukey’s tests were used. Results with a *p* value of less than 0.05 were considered to be significant. Data were presented as means ± SEM.

## 5. Conclusions

LRRC8A inhibition selectively restored sensitivity to DOX, GEM, and 5-FU, primarily by modulating canonical drug resistance pathways governed by multidrug resistance-associated transporters and cytochrome P450 metabolic enzymes. Mechanistically, LRRC8A inhibition downregulated MRP3 and CYP3A4 through the suppression of NRF2 activity and its downstream transcriptional regulators, CEBPB and CEBPD (Figure 17). Moreover, our findings identify AKT2 phosphorylation as a central signaling event that links LRRC8A blockade to NRF2 inactivation. Collectively, these results establish LRRC8A as a critical regulatory hub in chemoresistance within breast cancer spheroids and underscore its potential as a promising therapeutic target for overcoming multidrug resistance in tumor cells with stem-like properties.

Despite the strengths of this study, several limitations should be acknowledged. This study relies primarily on in vitro 3D spheroid models of two breast cancer cell lines, which cannot fully recapitulate the complex tumor microenvironment. In addition, validation in patient-derived organoids and in vivo xenograft models will be essential to establish the translational relevance of LRRC8A-dependent chemoresistance mechanisms. This study focuses on MRP3 and CYP3A4 as primary downstream determinants of LRRC8A-mediated chemoresistance. However, additional pathways, including metabolic rewiring, epigenetic modifications, and DNA damage responses, may contribute to resistance to DOX, GEM, and 5-FU. Continued efforts to elucidate the LRRC8A signaling in the tumor microenvironment will accelerate the development of novel therapeutic approaches aimed at eradicating drug-resistant, stem-like tumor cell populations in breast cancer.

## Data Availability

The original contributions presented in this study are included in the article/Supplementary Materials. Further inquiries can be directed to the corresponding author.

## Supplementary Materials

The following supporting information can be downloaded at: https://www.mdpi.com/article/10.3390/ijms27062646/s1.

## Abbreviations

2D/3D, two/three-dimensional; ABC, ATP-binding cassette; ACTB, β-actin; AKT, protein kinase B; ANO, anoctamin; CEBP, CCAAT/enhancer binding protein; CYP, cytochrome P450; DOX, doxorubicin; DTX, docetaxel; EDV, endovion; ERK, extracellular signal regulated kinase; FU, fluorouracil; GEM, gemcitabine; GSK, glycogen synthase kinase; KCZ, ketoconazole; KLF, Kruppel-like factor; LAT, L-amino acid transporter; LRRC, leucine-rich repeat-containing protein; MDR, multidrug resistance; miRNA, microRNA; MRP, multidrug resistance-associated protein; NOX, nicotinamide adenine dinucleotide phosphate (NADPH) oxidase; NRF, nuclear factor, erythroid 2-related factor; OX, oxaliplatin; PI3K, phosphoinositide 3-kinase; PTX, paclitaxel; SIRT, sirtuin; SLC, solute carrier family; WNK, with-no-lysine (K) kinase.
