# Supplementary material for: LRRC8A Inhibition Overcomes Chemoresistance by Downregulating MRP3 and CYP3A4 in the 3D Spheroid Model of Human Breast Cancer Cells
Source: Int J Mol Sci. 2026 Mar 13;27(6):2646. doi: 10.3390/ijms27062646 (PMC13026703; doi:10.3390/ijms27062646)
Supplement: Supplementary file 1 [file ijms-27-02646-s001.zip › ijms-4056207-supplementary.pdf]

Supplementary Materials

LRRC8A Inhibition Overcomes Chemoresistance by Downregulating MRP3 and CYP3A4 in the 3D Spheroid Model of Human Breast Cancer Cells

Ryo Otsuka [1,†], Junko Kajikuri [1,†], Miki Matsui [1], Hiroaki Kito [1], Ayano Kitahara [1], Hinako Mitsui [1], Yohei Yamaguchi [1], Tomoka Hisada [2], Tatsuya Toyama [2] and Susumu Ohya [1,*]

1 Department of Pharmacology, Graduate School of Medical Sciences, Nagoya City University, Nagoya 467-8601, Japan
2 Department of Breast Surgery, Graduate School of Medical Sciences, Nagoya City University, Nagoya 467-8601, Japan
* Correspondence: sohya@med.nagoya-cu.ac.jp; Tel.: +81-52-853-8149
† These authors contributed equally to this work.

Supplementary Materials

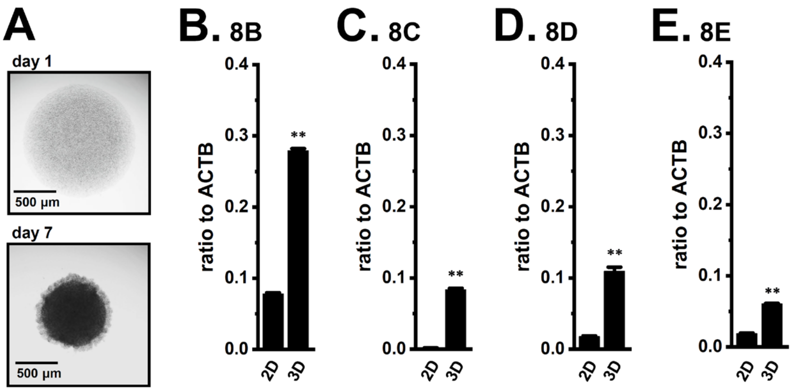

Figure S1. Phenotypic properties of YMB-1 cells cultured with ultra-low attachment plates and comparison of LRRC8 isoforms (LRRC8B, 8C, 8D, and 8E) expression between 2D monolayers and 3D spheroids of YMB-1 cells. (**A**): Phenotypic properties of YMB-1 cells cultured with PrimeSurface 96 U plates (upper panel: day 1, lower panel: day 7). The scale bar shows 500 μm. (**B-E**): Real-time PCR examination of the LRRC8B (**B**), LRRC8C (**C**), LRRC8D (**D**), and LRRC8E (**E**) transcripts in 2D monolayers and 3D spheroids. Expression levels are shown as a ratio to ACTB (n = 4). Results are expressed as means ± SEM. **: P <0.01 vs. 2D.

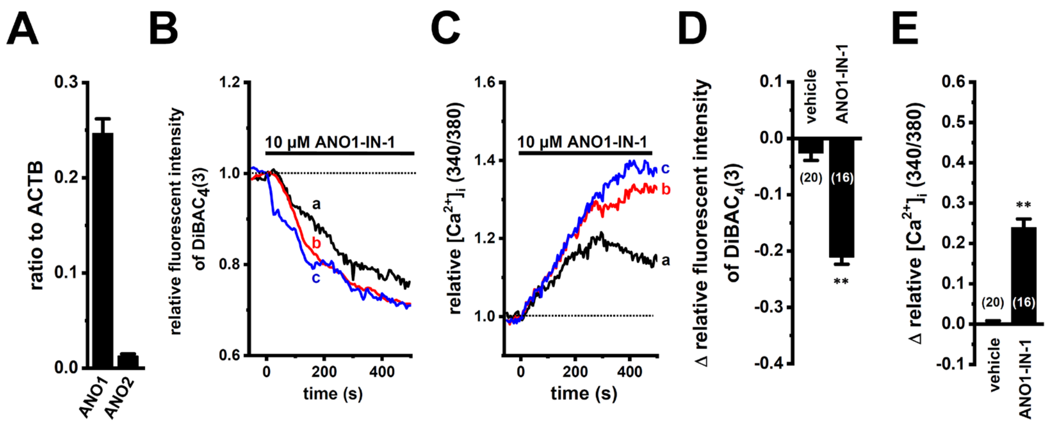

Figure S2. Functional expression of ANO1 in isolated cells from YMB-1 3D spheroids. (**A**): Real-time PCR examination of ANO1 and ANO2 in 3D spheroids. Expression levels are shown as a ratio to ACTB ($n = 4$). (**B, C**): Simultaneous measurement of changes in membrane potential (**B**) and $[Ca^{2+}]_i$ (**C**) in three different cells [a (black, b (red), and c (blue)], following the application of the selective ANO1 inhibitor, ANO1-IN-1 (10 μM). The relative time courses of changes in fluorescence intensities (1.0 at time 0 s) from isolated cells are shown. (**D, E**): Summarized results of ANO1-IN-1 (10 μM)-induced hyperpolarizing responses (**D**) and changes in $[Ca^{2+}]_i$ (**E**) at 6 min. Numbers used for experiments are shown in parentheses. Results are expressed as means ± SEM. **: P <0.01 vs. the vehicle control.

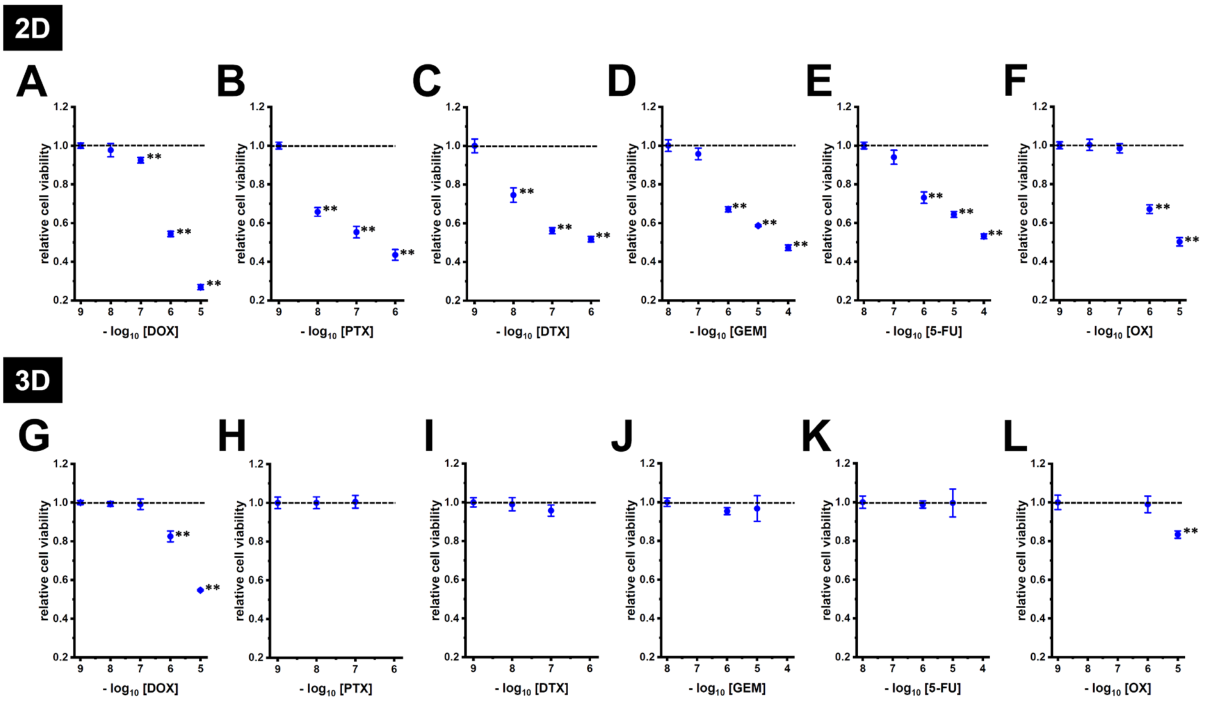

Figure S3. Concentration-response relationships of DOX, PTX, DTX, GEM, 5-FU, and OX for viability in 2D monolayers and 3D spheroids of YMB-1 cells. (**A-L**): Effects of treatment with DOX (**A, G**), PTX (**B, H**), DTX (**C, I**), GEM (**D, J**), 5-FU (**E, K**), and OX (**F, L**) for 48 hr on the viability of 2D- (**A-F**) and 3D- (**G-L**) cultured cells. Viability in vehicle-treated cells was expressed as 1.0. **: P <0.01 vs. the vehicle control.

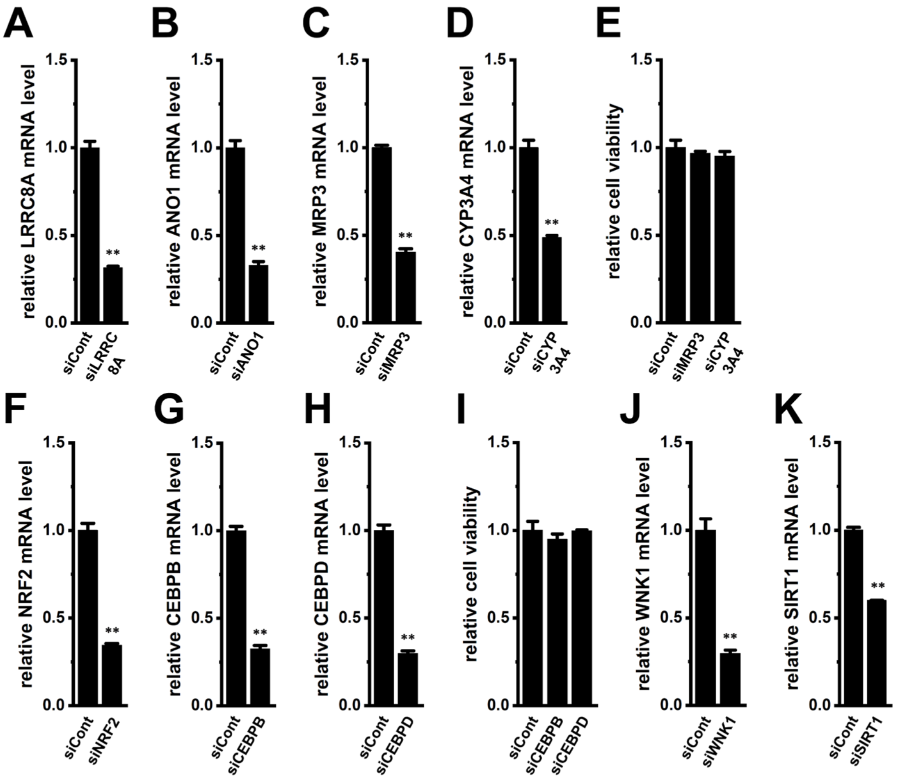

Figure S4. The transcriptional repression efficacy of siRNAs in YMB-1 3D spheroids and effects of the siRNA-mediated inhibition of MRP3, CYP3A4, CEBPB, and CEBPD on the cell viability. (**A-D, F-H, J-L**): Real-time PCR examination of LRRC8A (**A**), ANO1 (**B**), MRP3 (**C**), CYP3A4 (**D**), Nrf2 (**F**), CEBPB (**G**), CEBPD (**H**), WNK1 (**J**), and SIRT1 (**K**) in YMB-1 3D spheroids transfected with siCont, siLRRC8A (**A**), siANO1 (B), siMRP3 (**C**), siCYP3A4 (**D**), siNrf2 (**F**), siCEBPB (**G**), siCEBPD (**H**), siWNK1 (**J**), and siSIRT1 (**K**), respectively ($n = 4$). After normalization to ACTB mRNA levels, expression in the siCont group was expressed as 1.0. (**E, I**): Effects of the siRNA-mediated inhibition of MRP3, CYP3A4, CEBPB, and CEBPD on the viability of 3D spheroids using the WST-1 assay ($n = 5$). Cell viability in the siCont group is expressed as 1.0. Results are expressed as means ± SEM. **: $P < 0.01$ vs. siCont.

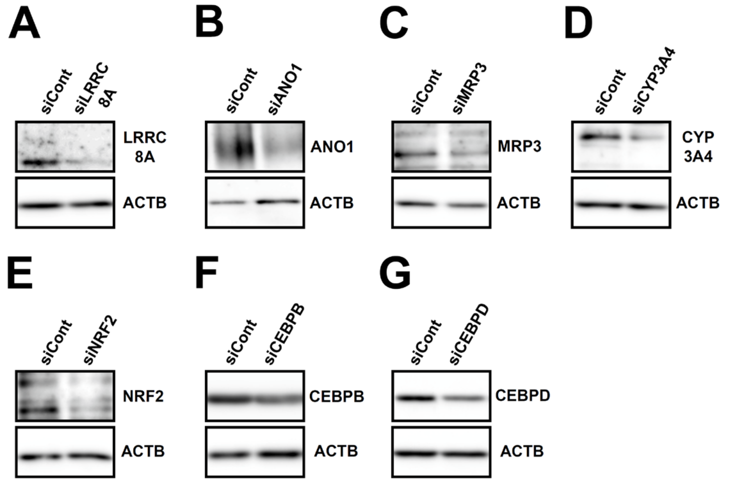

Figure S5. Reduced protein expression levels following siRNA transfection in YMB-1 3D spheroids. (**A-G**): Protein expression of LRRC8A (**A**), ANO1 (**B**), MRP3 (**C**), CYP3A4 (**D**), NRF2 (**E**), CEBPB (**F**), CEBPD (**G**) in YMB-1 3D spheroids following transfection with control siRNA (siCont) or the corresponding target-specific siRNAs (siLRRC8A, siMRP3, siCYP3A4, siCEBPB, siCEBPD, siNRF2, and siANO1. Immunoblots were probed with antibodies against LRRC8A, ANO1, MRP3, CYP3A4, NRF2, CEBPB, CEBPD, and ACTB.

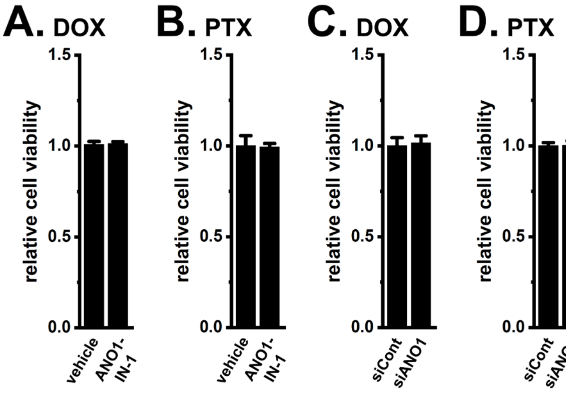

Figure S6. Effects of the pharmacological and siRNA-mediated inhibition of ANO1 on resistance to DOX and PTX in YMB-1 3D spheroids. (**A-D**): Effects of the treatment with 1 µM doxorubicin (DOX) (**A, C**) and 0.1 µM paclitaxel (PTX) (**B, D**) for 48 hr on the cell viability of YMB-1 spheroids pre-treated with vehicle and 10 µM ANO1-IN-1 for 24 hr (**A, B**) and transfected with negative control siRNA (siCont) and human ANO1 siRNA (siANO1) (**C, D**) (n = 5). Cell viability in the vehicle control and siCont groups was expressed as 1.0. Results are expressed as means ± SEM.

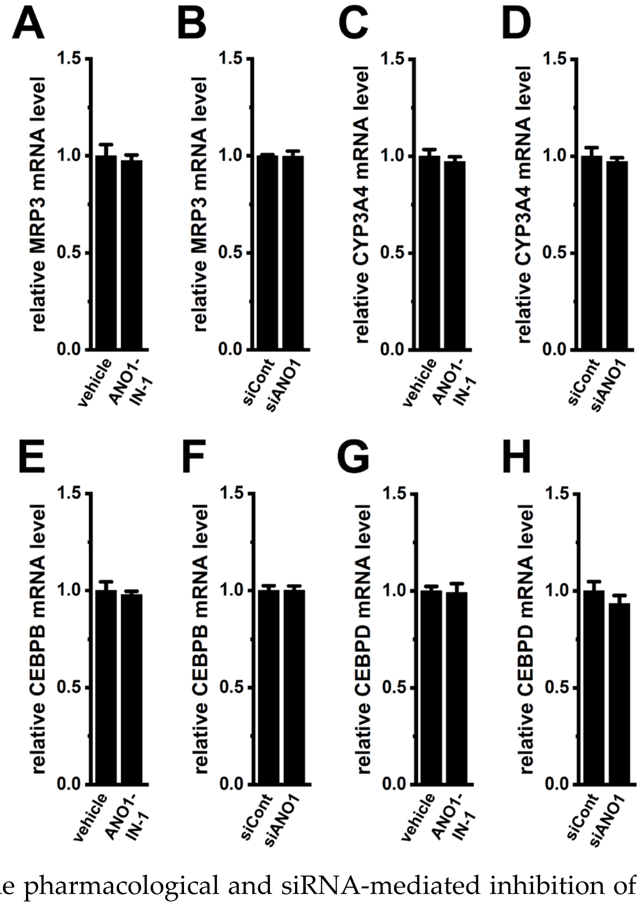

Figure S7. Effects of the pharmacological and siRNA-mediated inhibition of ANO1 on the expression of MRP3, CYP3A4, CEBPB, and CEBPD in YMB-1 3D spheroids. (**A-H**): Real-time PCR examination of MRP3 (**A, B**), CYP3A4 (**C, D**), CEBPB (**E, F**), and CEBPD (**G, H**) in YMB-1 3D spheroids treated with vehicle and ANO1-IN-1 (10 µM) for 12 hr (**A, C, E, G**) and transfected with siCont and siANO1 (**B, D, F, H**). After normalization to ACTB mRNA expression levels, mRNA levels in the vehicle control and siCont groups were expressed as 1.0. Results are expressed as means ± SEM.

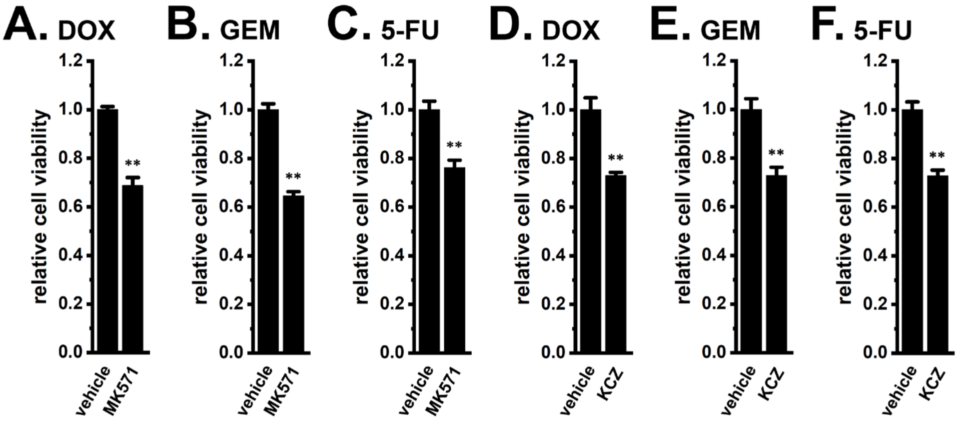

Figure S8. Effects of the pharmacological inhibition of MRPs and CYP3A4 on resistance to DOX, GEM, and 5-FU in YMB-1 3D spheroids. (**A-F**): Effects of the treatment with 1 μM DOX (**A, D**), 10 μM GEM (**B, E**), and 10 μM 5-FU (**C, F**) for 48 hr on the cell viability of YMB-1 spheroids co-treated with vehicle, 20 μM MK571, a pan-MRP inhibitor (**A-C**), and 0.1 μM ketoconazole (KCZ), a potent CYP3A4 inhibitor (**D-F**) using the WST-1 assay ($n = 5$). Viability in the untreated group was expressed as 1.0. Results are expressed as means ± SEM. **: $P < 0.01$ vs. the vehicle control.

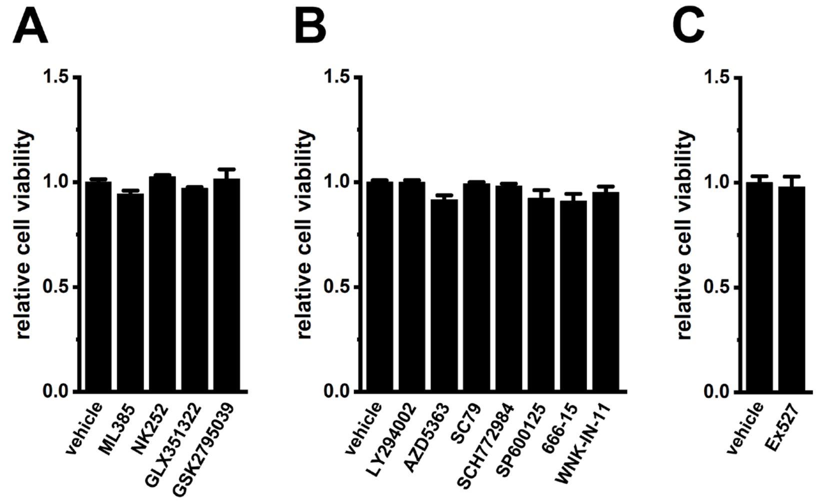

Figure S9. Non-toxic effects of compounds used in Sections 2.5., 2.6., 2.7., and 2.9. on the viability of YMB-1 3D spheroids. (**A-C**): Effects of ML385 (5 μM), NK252 (100 μM), GLX351322 (10 μM), GSK2795039 (10 μM), LY294002 (10 μM), AZD5363 (2 μM), SC79 (10 μM), SCH772984 (1 μM), SP600125 (1 μM), 666-15 (1 μM), WNK-IN-11 (1 μM), and Ex527 (1 μM) on the viability of YMB-1 3D spheroids. Cells were treated for 12 hr (**A, B**) or 24 hr (**C**), and viability was assessed using the WST-1 assay ($n = 5$). The viability of the vehicle control-treated cells was normalized to 1.0. Results are expressed as means ± SEM.

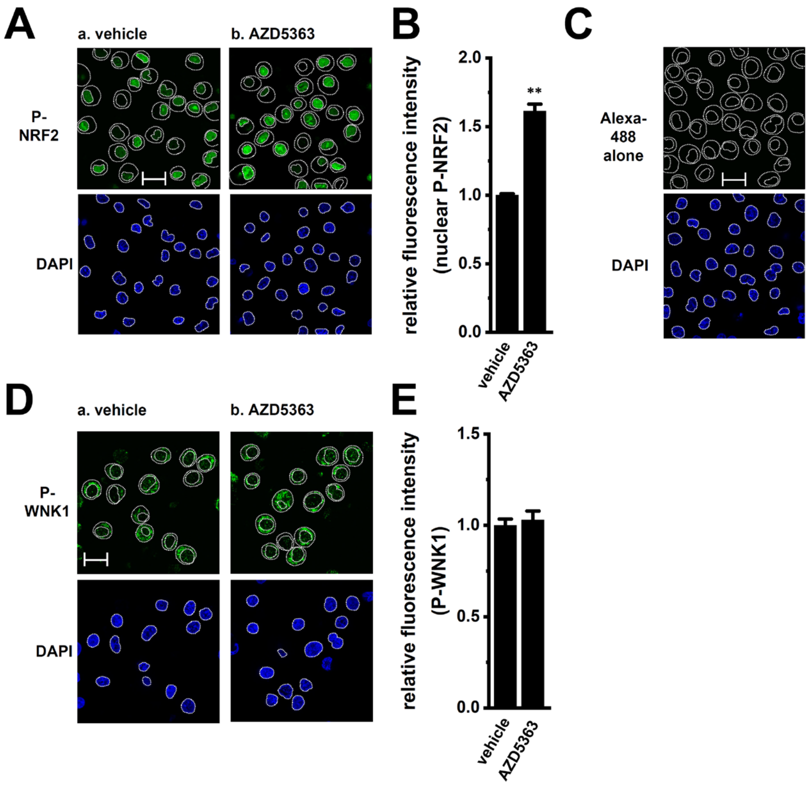

Figure S10. Effects of AKT inhibition on the nuclear translocation of P-NRF2 and cellular distribution of P-WNK1 in isolated cells from YMB-1 3D spheroids. (**A, B**): Confocal fluorescent images of Alexa Fluor 488-labeled P-NRF2 in vehicle- (**a**) and AZD5363 (2 μM) (**b**)-treated cells isolated from YMB-1 spheroids for 2 hr (**A**). Nuclear morphologies were shown by DAPI images. Dashed lines show the plasma membrane and nuclear boundary. Summarized results of the mean Alexa Fluor 488 fluorescence intensities in the nuclei (*n* = 6) (**B**). In each batch (*n* = 1), more than 20 cells treated with vehicle and AZD5363 were observed by confocal laser scanning microscopy. (**C**): Confocal fluorescent images of Alexa Fluor 488 alone in untreated cells isolated from YMB-1 spheroids. (**D, E**): Confocal fluorescent images of Alexa Fluor 488-labeled P-WNK1 in vehicle- (**a**) and AZD5363 (**b**)-treated cells isolated from YMB-1 spheroids for 2 hr (**D**). Summarized results of the mean fluorescence intensities Alexa Fluor 488 in whole cells (*n* = 6) (**E**). The scale bar shows 20 μm. Results are expressed as means ± SEM. **: *P* <0.01 vs. the vehicle control.

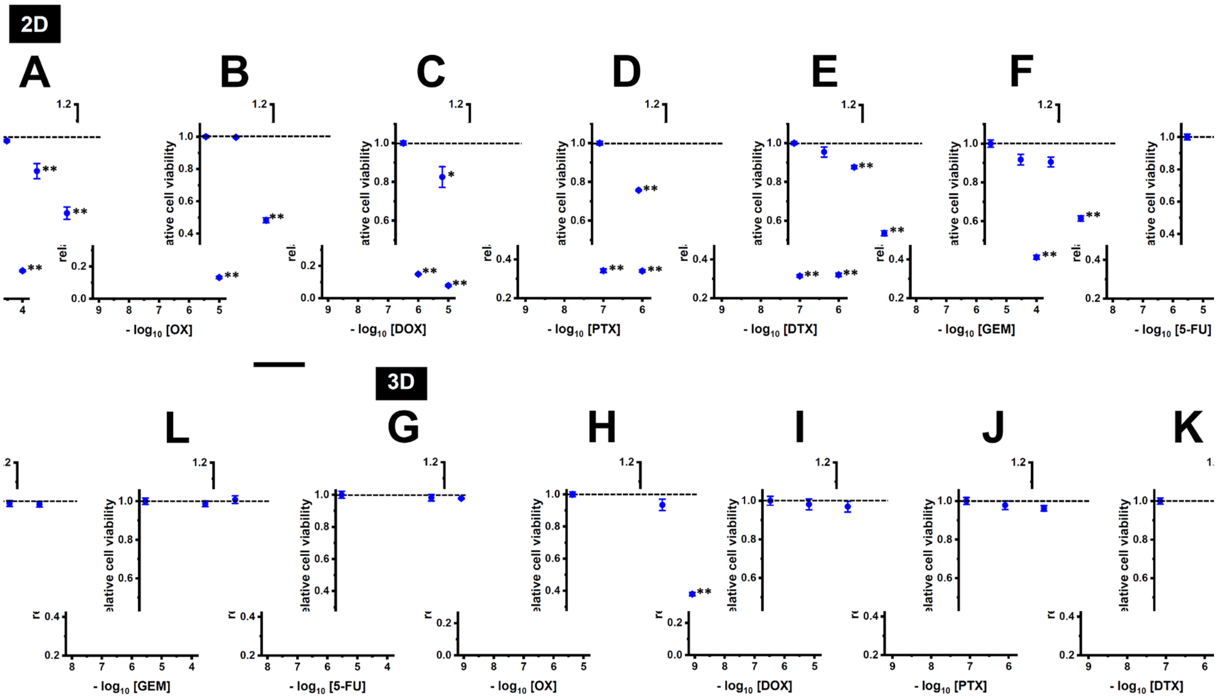

Figure S11. Concentration-response relationships of DOX, PTX, DTX, GEM, 5-FU, and OX for viability in 2D monolayers and 3D spheroids of MDA-MB-468 cells. (**A-L**): Effects of treatment with DOX (**A, G**), PTX (**B, H**), DTX (**C, I**), GEM (**D, J**), 5-FU (**E, K**), and OX (**F, L**) for 48 hr on the viability of '2D' (**A-F**) and '3D' (**G-L**). The viability of the vehicle control-treated cells was expressed as 1.0. **: P <0.01 vs. the vehicle control.

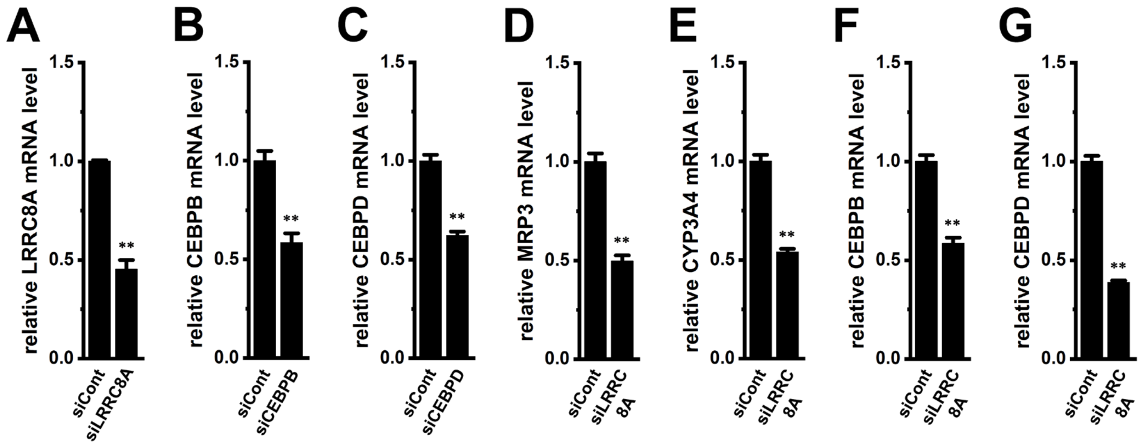

Figure S12. The transcriptional repression efficacy of siRNAs in MDA-MB-468 3D spheroids and effects of the siRNA-mediated inhibition of LRRC8A on the expression of MRP3, CYP3A4, CEBPB, and CEBPD transcripts in MDA-MB-468 3D spheroids. (**A-C**): Real-time PCR examination of LRRC8A (**A**), CEBPB (**B**), and CEBPD (**C**) in MDA-MB-468 3D spheroids transfected with siCont, siLRRC8A (**A**), siCEBPB (**B**), and siCEBPD (**C**) for 48 hr, respectively (n = 4). (**D-G**): Real-time PCR examination of MRP3 (**D**), CYP3A4 (**E**), CEBPB (**F**), and CEBPD (**G**) in MDA-MB-468 3D spheroids transfected with siCont and siLRRC8A, respectively (n = 4). After normalization to ACTB mRNA levels, expression in the siCont group was expressed as 1.0. Results are expressed as means ± SEM. **: P < 0.01 vs. siCont.

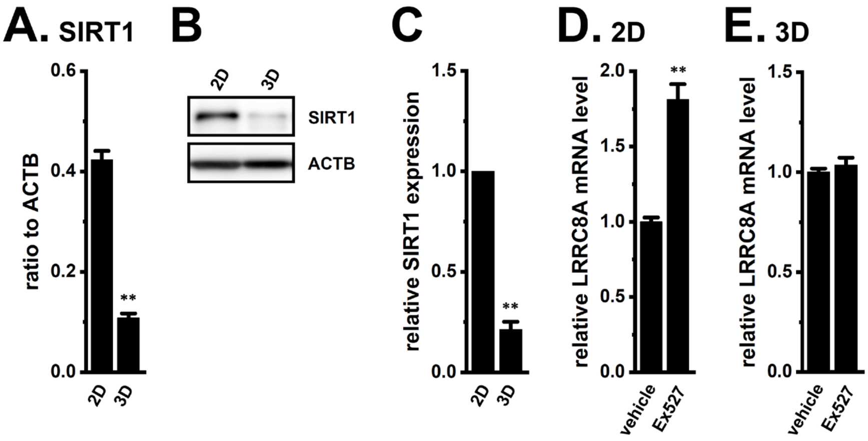

Figure S13. SIRT1-mediated transcriptional regulation of LRRC8A during 3D spheroid formation of YMB-1 cells. (**A**): Real-time PCR examination of SIRT1 in 2D monolayers and 3D spheroids of YMB-1 cells. Expression levels are shown as a ratio to ACTB ($n = 4$). (**B, C**): Protein expression of SIRT1 in '2D' and '3D' groups. Blots were probed with anti-SIRT1 (upper panel) and anti-ACTB (lower panel) antibodies (**B**). Summarized results were obtained as the optical density of SIRT1 and ACTB band signals. After compensation for the optical density of the SIRT1 protein band signal with that of the ACTB signal, the optical density in '2D' was expressed as 1.0 ($n = 4$) (**C**). (**D, E**): Effects of the pharmacological inhibition of SIRT1 with Ex527 (1 μM) for 12 hr on the expression level of LRRC8A transcripts in '2D' (**D**) and '3D' (**E**). Expressions in the vehicle control was expressed as 1.0 ($n = 4$). **: P <0.01 vs. 2D and vehicle control.

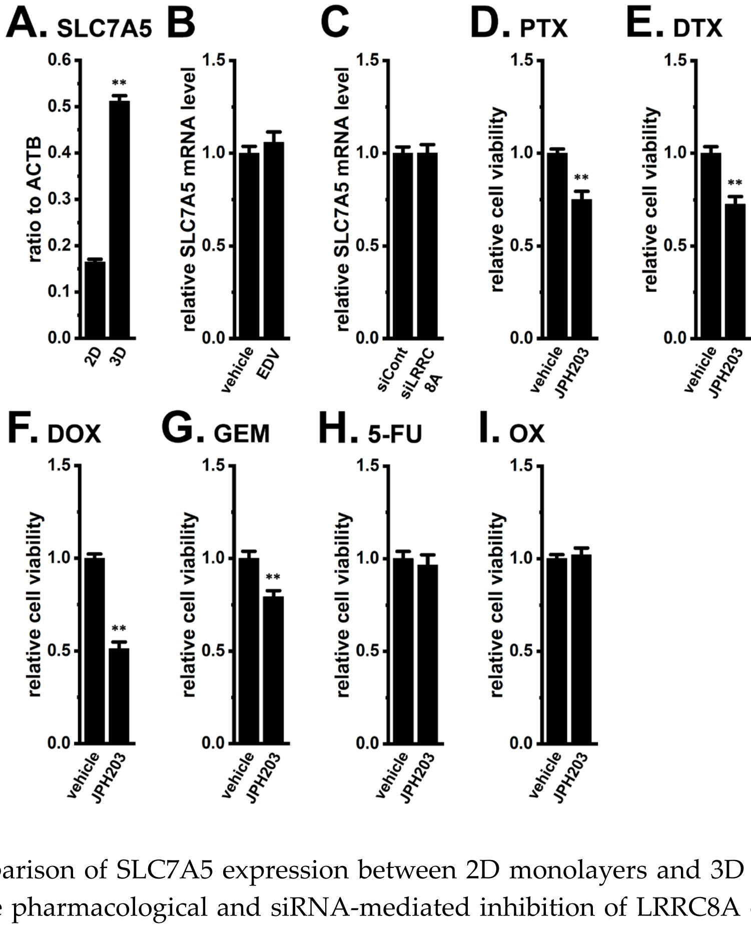

Figure S14. Comparison of SLC7A5 expression between 2D monolayers and 3D spheroids of YMB-1 cells, effects of the pharmacological and siRNA-mediated inhibition of LRRC8A on the expression of SLC7A5 transcripts, and effects of the SLC7A5 inhibition on chemoresistance in YMB-1 3D spheroids. (**A**): Real-time PCR examination of SLC7A5 in 2D monolayers and 3D spheroids of YMB-1 cells.

Expression levels are shown as a ratio to ACTB (n = 4 for each). (**B, C**): Real-time PCR examination of SLC7A5 in YMB-1 3D spheroids treated with vehicle and 10 μM EDV for 12 hr (**B**) and transfected with siCont and siLRRC8A (**C**). After normalization to ACTB mRNA expression levels, the SLC7A5 mRNA expression levels in the vehicle control and siCont groups are expressed as 1.0. (**D-I**): Effects of the treatment with vehicle, 0.1 μM PTX (**D**), 0.1 μM DTX (**E**), 1 μM DOX (**F**), 10 μM GEM (**G**), 10 μM 5-FU (**H**), and 10 μM OX (**I**) for 48 hr on the cell viability of YMB-1 spheroids co-treated with vehicle and 10 μM JPH203, a SLC7A5 inhibitor, using the WST-1 assay (n = 5). Cell viability in the untreated group was expressed as 1.0. Results are expressed as means ± SEM. **: P <0.01 vs. 2D and vehicle control.

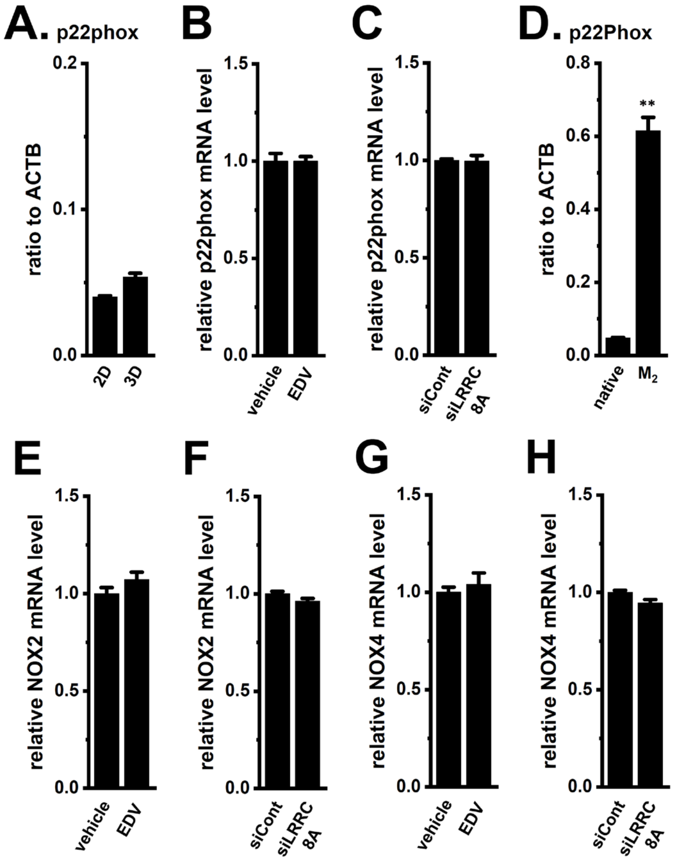

Figure S15. Comparison of p22phox expression between 2D monolayers and 3D spheroids of YMB-1 cells and effects of the pharmacological and siRNA-mediated inhibition of LRRC8A on the expression of p22phox transcripts in YMB-1 3D spheroid. (**A**): Real-time PCR examination of p22phox in 2D monolayers and 3D spheroids of YMB-1 cells. (**B, C**): Real-time PCR examination of p22phox in YMB-1 3D spheroids treated with vehicle and 10 μM EDV for 12 hr (**B**) and transfected with siCont and siLRRC8A (**C**). (**D**): Real-time PCR examination of p22phox in native THP-1 cells and THP-1-differentiated M_2 macrophages. (**E-H**): Real-time PCR examination of NOX2 (**E, F**) and NOX4 (**G, H**) in YMB-1 3D spheroids treated with vehicle and 10 μM EDV for 12 hr (**E, G**) and transfected with siCont and siLRRC8A (**F, H**). In 'A' and 'D', expression levels are shown as a ratio to ACTB (n = 4 for each). In addition, after normalization to ACTB mRNA levels, the p22phox, NOX2, and NOX4 mRNA expression levels in the vehicle control and siCont groups are expressed as 1.0. Results are expressed as means ± SEM. **: P < 0.01 vs. native THP-1.

Table S1. List of PCR primers used in this study, related to Section 4.3.

Target name	Genbank accession number	Primer sequence		Amplicon length (bp)
		Forward (5' to 3')	Reverse (5' to 3')	
CD24	NM_013230	GCTGCTGGCACTGCTCCTA	GGTGGTGGCATTAGTTGGATTT	124
NANOG	NM_024865	CCTTCCTCCATGGATCTGCTTA	CTTGACCGGGACCTTGTCTTC	120
KLF4	NM_001314052	ACGGCTGTGGATGGAAATTC	GGTGGTCCGACCTGGAAAAT	120
LRRC8A	NM_001127244	ACCCCAACTCCACCATTCTG	ACCAGTGCAGTCGGTTCTCAT	120
MDR1	NM_001348945	TCACCATGGATGAGATTGAGAAA	TGGCGATCCTCTGCTTCTG	128
MRP1	NM_004996	AGGCGAGTGTCTCCCTCAAA	TCCTCACGGTGATGCTGTTC	120
MRP2	NM_000392	CTCAAGAACCTGCTGGCCTTT	ATTGTTGCTATCCACCAGGACAT	120
MRP3	NM_003786	GGCTGGGCTGATGTTCCTG	GCCTTCCTGTAGATGACACCCATG	123
MRP4	NM_005845	CTGTGCGGCTGACGGTTAC	GGTTGCGCTGTGATATCTCATC	120
MRP5	NM_005688	CCCAGGCAACAGAGTCTAACCT	CACCGGTTCGGTAATTCAAT	120
MRP6	NM_001171	TTAGACGCGAGAGGTCCATCA	CGTATTGGATGCTGTCCTTTCC	129
MRP7	NM_001198934	TCGTGGGTGGTCTCATCTTG	GCTTAACCCGCGCATCCT	114
ABCG1	NM_016818	AGGGACTCGGTCCTGACACA	GAAGCCGGAGTTGCTCAAGA	120
ABCG2	NM_004827	CGATATGGATTTACGGCTTTGCA	CCAAATATTCTTCGCCAGTACATG	121
MDR3	NM_000443	CCACAGATGCTGCCCAAGT	GGGTTAACTGCCAACCGTAGAT	120
ABCB5	NM_001163941	GTCTCAATGGCAGTGGGAAGA	GATAATGCCGCACATTTAAAGCT	120
ABCB10	NM_012089	TCAGGATTCCCTGGCACAAG	TGGTCCACTTTGCTGGCATA	120
CYP1B1	NM_000104	CGACATGATGGACGCCTTTA	GCGCCGAAGATGTCAGTGAT	120
CYP2B6	NM_000767	GCCCTCCAGAGCTTCATGAC	TGGTGTGTTGGGTGACAATGT	120
CYP3A4	NM_017460	CCACAAAGCTCTGTCCGATCT	TCAGGGTGAGTGGCCAGTTC	120
CYP2A6	NM_000762	TCGTGGTGCTGTGTGGACAT	ATACCTCGCCATAGCCTTTGA	120
CYP2A7	NM_000764	AGGAGTCGGGCTTCCTCATC	AGCGGTCCCCAAAGACAAT	120
CYP3A5	NM_000777	AAGAAAAGTCGCCTCAACGAC	GCGAGCTCCAGATCAGACAGA	119
CYP2C8	NM_000770	GGCCCCACTCCTCTTCCTAT	CATGCCAAAATACACGGTGAA	120
CYP2D6	NM_000106	ACCTAGCTCAGGAGGGACTGAA	AAGCCTTTTGGAAGCGTAGGA	120
CYP4Z1	NM_178134	CCTTGTGGGTTGGACCCTTT	CCCAGGATTCAAGGATTTTGTG	120
NRF2	NM_006164	TGCCCCTGGAAGTGTCAAAC	TCACATTGGGCATCATGCA	120
CEBPA	NM_001287435	CCAGGCGGTGGCTTCTC	GGCATTGGAGCGGTGAGTT	121
CEBPB	NM_001285878	GCCCTCGCAGGTCAAGAG	TGCGCACGGCGATGT	107
CEBPE	NM_001805	CAAGGGCAAGAAGGCAGTGA	TGCTGCGTCTCCAGAATGC	120
CEBPD	NM_005195	TCCTGTTGATGCAGCTAAGGTACA	GCATGCTCAGTCTTTTCCTCTTATC	100
CEBPG	NM_001806	ACGCCGAGAGAGGAACAACA	TTTGCTTCCAACCGTTCATTC	120
CEBPZ	NM_001195053	GCAAGAGGTCCTGTCTTCAGATG	TCAGTCAGCCAAGCCAGAGA	120
NOX1	NM_007052	ATTCACGGCATTGGTGGAA	GCGCCTACAGTGGGAGTCA	120
NOX2	NM_000397	CGGGACACACATGCCTTTG	CTCATCCCAGCCAGTGAGGTA	120
NOX3	NM_01571	TCGAGGCCAAACCCAAGAC	GAGGGTTCCTTGCCAGAAAAT	120
NOX4	NM_016931	CTGGACCTTTGTGCCTGTACTG	TGACCATTCGGATTTCCATGA	120
NOX5	NM_02450	GGCATCACCCCCTTTGCT	TGGAGCTTCATGTTGTCTTGGA	120
Duox1	NM_017434	GCGGCCTCTGAGCAGTTC	CTCACGGCTCCAGTAGCTGTT	144
Duox2	NM_014080	GGGTCTGCAACAACTACTGGATTC	CAACCACTATGTTGTCCTCCAACT	120
LRRC8B	NM_015350	CAGCTCCATTGGTTTGCAAA	AAAATGCTCGAGCCTGGAACT	120
LRRC8C	NM_032270	AAGGCCTGAAGACAGATTTGGA	CCAGGGTATGGATGAGGACAA	120
LRRC8D	NM_001134479	TGCCCGATGCTGTCTTTGA	GCAGAGGTGGAGCTCTTGGA	117
LRRC8E	NM_025061	TCTTCTCCAAGCTGGCCTTCT	GCACGGAACGGAAGGAGTAC	120
SIRT1	NM_012238	ATTTTCCATGGCGCTGAGGTA	CCTCCATGGGTTCTTCTAAACTTG	121
ANO1	NM_018043	TCCACGGAGTCGGGTTTGT	GCCACGGGTCTCATTAATGTG	120
ANO2	NM_001278596	CGCAGCCAGAAAACTCACAGT	ACGGGCAGACAGAATAAACCA	128
WNK1	NM_018979	AGCAGCTGCCACCTTTTCC	CAACCGCAGAAGTCACTGTGA	120
SLC7A5	NM_003486	CGCCTTCTCCAAGGACATCTT	CGCTCAAGCTCAGGCTTTCT	120
p22phox	NM_000101	CTTTGGTGCCTACTCCATTGTG	ACCACGGCGGTCATGTACTT	120
ACTB	NM_001101	AGGCCAACCGCGAGAAGATG	GCCAGAGGCGTACAGGGATA	101
hsa-miR17-5p		GCAAAGTGCTTACAGTGCAG		
hsa-miR93-5p		GCAAAGTGCTGTTCGTGC		
U6		CTCGCTTCGGCAGCACA	AACGCTTCACGAATTTGCGT	

Table S2. List of antibodies used in this study, related to Sections 4.4. and 4.8.

Type	Antibody name	Host species	Working dilution	Company	Product code	Observed MW (approx. kDa)
Primary antibody	Phospho-NRF2 (Ser40)	rabbit monoclonal	1:100 (ICC)	Abclonal (Tokyo, Japan)	AP1133	
	NRF2	rabbit polyclonal	1:100 (ICC)	Abclonal	A21508	
	NRF2	rabbit polyclonal	1:2000 (WB)	Proteintech (Rosemont, IL, USA)	16396-1-AP	110
	LRRC8A	rabbit polyclonal	1:2000 (WB)	Abcepta (San Diego, CA, USA)	AP19519b	95
	ANO1	rabbit polyclonal	1:1500 (WB)	Abclonal	A110498	150
	MRP3	rabbit polyclonal	1:800 (WB)	Abclonal	A9849	150
	CYP3A4	rabbit polyclonal	1:2400 (WB)	Proteintech	18227-1-AP	65
	CEBPB	rabbit polyclonal	1:20000 (WB)	Proteintech	23431-1-AP	36
	CEBPD	rabbit polyclonal	1:2000 (WB)	Proteintech	23895-1-AP	43
	NOX2	rabbit polyclonal	1:100 (ICC)	Proteintech	19013-1-AP	
	NOX4	rabbit polyclonal	1:100 (ICC)	Proteintech	14347-1-AP	
	Phospho-AKT1 (Ser473)	rabbit polyclonal	1:1000 (WB)	BioLegend (San Diego, CA, USA)	649001	60
	AKT1	mouse monoclonal	1:1200 (WB)	BioLegend	680302	60
	Phospho-AKT2 (Ser474)	rabbit monoclonal	1:800 (WB)	Cell Signaling Technology (Tokyo, Japan)	8599	60
	AKT2	rabbit polyclonal	1:1000 (WB)	Proteintech	28113-1-AP	60
	Phospho-GSK3B (Ser9)	rabbit monoclonal	1:800 (WB)	Cell Signaling Technology	9323	45
	GSK3B	rabbit polyclonal	1:4000 (WB)	Proteintech	22104-1-AP	45
	SIRT1	rabbit polyclonal	1:1500 (WB)	MBL (Nagoya, Japan)	CY-P1016	130
	Phospho-WNK1 (Thr60)	rabbit monoclonal	1:600 (WB)<br>1:100 (ICC)	R&D Systems (Minneapolis, MN, USA)	MAB4720	280
	WNK1	rabbit polyclonal	1:2000 (WB)	Proteintech	28357-1-AP	280
	ACTB	mouse monoclonal	1:15000 (WB)	Sigma-Aldrich (St Louis, MO, USA)	A1978	43
	ACTB	rabbit polyclonal	1:1000 (WB)	MBL	PM053	43
Secondary antibody	HRP-conjugated anti-mouse IgG	goat polyclonal	1:15000 (WB)	ThermoFisher Scientific (Waltham, MA, USA)	31430	
	HRP-conjugated anti-rabbit IgG	goat polyclonal	1:7500 (WB)	ThermoFisher Scientific	AP307P	
	Alexa Fluor 488-labeled anti-rabbit IgG	goat polyclonal	1:1000 (ICC)	Abcam (Cambridge, UK)	ab150077	

WB: western blotting, ICC: immunocytochemistry, MW: molecular weight, HRP: horseradish peroxidase; MBL: Medical & Biological Laboratories
